# Optical signatures of interlayer electron coherence in a bilayer semiconductor

**DOI:** 10.1038/s41567-025-02971-0

**Published:** 2025-08-20

**Authors:** Xiaoling Liu, Nadine Leisgang, Pavel E. Dolgirev, Alexander A. Zibrov, Jiho Sung, Jue Wang, Takashi Taniguchi, Kenji Watanabe, Valentin Walther, Hongkun Park, Eugene Demler, Philip Kim, Mikhail D. Lukin

**Affiliations:** 1 Department of Physics, Harvard University, Cambridge, MA USA; 2 Department of Chemistry and Chemical Biology, Harvard University, Cambridge, MA USA; 3 Research Center for Materials Nanoarchitectonics, National Institute for Materials Science, Tsukuba, Japan; 4 Research Center for Electronic and Optical Materials, National Institute for Materials Science, Tsukuba, Japan; 5 Department of Physics and Astronomy, Purdue University, West Lafayette, IN USA; 6 Department of Chemistry, Purdue University, West Lafayette, IN USA; 7 Institute for Theoretical Physics, ETH Zurich, Zurich, Switzerland; 8 John A. Paulson School of Engineering and Applied Sciences, Harvard University, Cambridge, MA USA

**Keywords:** Two-dimensional materials, Optical spectroscopy, Condensed-matter physics

## Abstract

Emergent strongly correlated electronic phenomena in atomically thin transition-metal dichalcogenides are an exciting frontier in condensed matter physics, with examples ranging from bilayer superconductivity and electronic Wigner crystals to the ongoing search for exciton condensation. Here we take a step towards the latter by reporting experimental signatures of unconventional hybridization of the excitons with opposing dipoles consistent with coherence between interlayer electrons in a transition-metal dichalcogenide bilayer. We investigate naturally grown MoS_2_ homobilayers integrated in a dual-gate device structure allowing independent control of the electron density and out-of-plane electric field. By electron doping the bilayer when electron tunnelling between the layers is negligible, we observe that the two interlayer excitons hybridize, displaying unusual behaviour distinct from both conventional level crossing and anti-crossing. We show that these observations can be explained by quasi-static random coupling between the excitons, which increases with electron density and decreases with temperature. We argue that this phenomenon is indicative of a spatially fluctuating order parameter in the form of interlayer electron coherence, a theoretically predicted many-body state that has yet to be unambiguously established experimentally outside of the quantum Hall regime.

## Abstract (web summary)

Condensates of excitons have been observed in the quantum Hall regime, but evidence for their existence at low magnetic fields remains controversial. Now evidence of coherence between optically pumped interlayer excitons in MoS_2_ marks a step towards confirming exciton condensation at low magnetic fields.

## Main

**Fig. 1:**
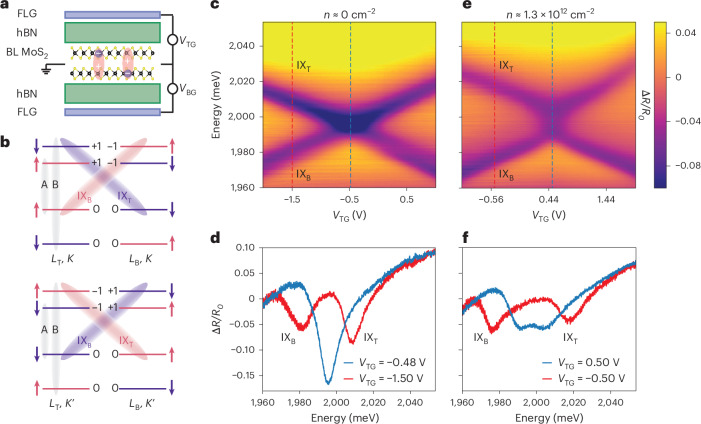
The d.c. Stark effect of interlayer excitons. **a**, A schematic of a dual-gated 2*H*-stacked MoS_2_ homobilayer (BL MoS_2_) encapsulated with hBN. Tuning of the top and bottom gates, composed of a few layers of graphene (FLG), allows independent control of the total electron density *n* and out-of-plane electric field *E*_*z*_. Interlayer excitons (IXs), highly sensitive to *E*_*z*_ owing to their large dipole moments, are also depicted. **b**, A schematic of the electronic band structure near the *K* valleys (top) and ${K}^{{\prime} }$ valleys (bottom) showing the relevant excitonic levels, electron spin and corresponding AQNs of the electronic bands, which determine optical selection rules. Top and bottom layers are labelled as *L*_T_ and *L*_B_, respectively. **c**, In the undoped case *n* = 0, the energies of interlayer excitons shift linearly with *E*_*z*_ (*V*_BG_ = −1.15*V*_TG_ − 1 V), as can be seen in the simple crossing of exciton branches in the measured reflectance map Δ*R*/*R*_0_ at *T* = 8 K. **d**, The system exhibits two well-separated branches at a finite *E*_*z*_ ≠ 0, becoming degenerate at *E*_*z*_ = 0, with doubled oscillator strength. **e**, The d.c. Stark effect for the doped sample with *n* ≈ 1.3 × 10^12^ cm^−2^ (*V*_BG_ = −1.15*V*_TG_ + 1.25 V), showing that the simple crossing in **c** turns into a stochastic avoided crossing (Fig. 2). **f**, The linecut at *V*_TG_ = 0.50 V, corresponding to *E*_*z*_ = 0, displays a broad feature with reduced relative amplitude compared with the undoped case in **d**. Source data

Transition-metal dichalcogenides (TMDs) are direct-gap semiconductors that can host optically bright excitons corresponding to Coulomb-bound electron–hole pairs. Due to the two-dimensional nature of TMDs, along with the large effective masses of electrons and holes and small dielectric permittivity of the surrounding medium, excitons are tightly confined, with the Bohr radius substantially smaller than the typical separation between doped charges^1^. These features make excitons in TMDs a promising tool for optical probing of many-body electron correlations. In particular, understanding the exciton fine structure of a doped sample has proven pivotal for a number of recent discoveries. Examples range from investigating polaronic dressing effects, which manifest through exciton line splitting into attractive and repulsive branches^2^, to probing correlated many-body phases using excited-state spectroscopy^3^, to observing electron crystalline states via umklapp scattering^4^ and to studying the rich magnetic properties of TMDs^5–7^. While most prior studies have focused on intralayer excitons, where both the exciton electron and hole reside in the same TMD layer, bilayer TMDs can host interlayer excitons (Fig. 1a,b), where the electron and hole are separated across the two layers^8^. However, interlayer excitons typically have weak optical transition dipole moments, posing challenges for optical measurements. In materials like MoS_2_ homobilayers, intra- and interlayer excitons strongly hybridize^9–11^, making interlayer excitons optically bright and enabling their use for optical probing of electronic correlations.

Here, we experimentally investigate the properties of indirect excitons in a naturally grown 2*H*-stacked MoS_2_ homobilayer, integrated into a dual-gate device structure (Fig. 1a) whereby the top and bottom gate voltages, *V*_TG_ and *V*_BG_, are simultaneously used to independently control the out-of-plane electric field *E*_*z*_ and the electron density *n* in the sample (Methods). The interlayer excitons have large permanent electric dipole moments ± *d*_*z*_ (Fig. 1a), which make them highly sensitive to *E*_*z*_. This can be studied by measuring reflectance contrast spectra (*R* − *R*_0_)/*R*_0_ = Δ*R*/*R*_0_ using a weak (optical nonlinearities are not relevant), incoherent white light source, where *R* is the reflectance obtained on the bilayer MoS_2_ flake and *R*_0_ is the reference spectrum at a high doping level (Methods). Figure 1c shows the undoped case (*n* = 0), illustrating the d.c. Stark effect, where the two interlayer excitons with opposite dipoles shift linearly with *E*_*z*_ and cross at *E*_*z*_ = 0 (*V*_TG_ ≈ −0.48 V). The degeneracy point *E*_*z*_ = 0 is characterized by the amplitude doubling in the reflectance contrast spectrum of interlayer excitons, Fig. 1d (blue curve). When the sample is doped (*n* ≈ 1.3 × 10^12^ cm^−2^), as extracted from simulations based on a simple capacitance model in Supplementary Section I, the simple crossing in Fig. 1c turns into the elongated shape shown in Fig. 1e. This effect is highly reproducible across different collection light spots within the same sample, as well as in other similar devices (Supplementary Section II). The putative degeneracy point *E*_*z*_ = 0 (*V*_TG_ ≈ 0.50 V), no longer exhibits the amplitude doubling (Fig. 1f). Instead, we observe a broadened feature with the overall amplitude roughly the same as that of individual interlayer excitons.

To understand these observations, we consider a simple model of two coupled harmonic oscillators describing the excitonic polarization response to the probe a.c. electric field ${\mathcal{E}}(t)$:

1 $$i\hslash {\partial }_{t}{X}_{{\rm{T}}}={\omega }_{{\rm{T}}}{X}_{{\rm{T}}}-i{\gamma }_{{\rm{T}}}{X}_{{\rm{T}}}+{\mathcal{W}}{X}_{{\rm{B}}}-{d}_{{\rm{T}}}{\mathcal{E}}(t),$$

and a similar equation holds for *X*_B_. Here, the variable *X*_T/B_ represents the polarization oscillations associated with the interlayer exciton IX_T/B_ (Fig. 1b), with the subscript referring to the layer of the electron; *ω*_T/B_ = ±*d*_*z*_*E*_*z*_ is the energy relative to the degeneracy point *E*_*z*_ = 0; *γ*_T/B_ is the total respective exciton decay rate; *d*_T/B_ refers to the corresponding transition dipole moment; and ${\mathcal{W}}$ is the coupling strength between the two interlayer excitons, which we introduced for reasons that will become clear shortly. Physically, this coupling can be viewed as a permanent dipole flip-flop process.

**Fig. 2:**
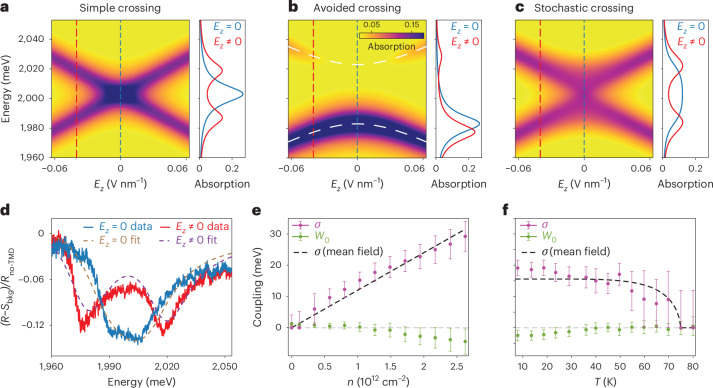
Stochastic interlayer exciton hybridization. **a**–**c**, The simulated absorption map exhibits a simple crossing (**a**) as in Fig. 1c when the two excitons are uncoupled (${{\mathcal{W}}}_{0}=0,\,\sigma =0$ in equation (2)), an avoided crossing (**b**) with asymmetry in the intensities of the two branches when the excitons are hybridized (${{\mathcal{W}}}_{0}=-20\,\,\text{meV}\,,\,\sigma =0$) and a stochastic crossing (**c**) reminiscent of Fig. 1e when the exciton coupling has a static, random character (${{\mathcal{W}}}_{0}=0,\,\sigma =20\,\text{meV}$). **d**, The measured reflectance contrast spectra are analysed using a few-parameter fit based on the model of stochastic coupling in equation (2); shown are two linecuts at *n* ≈ 1.2 × 10^12^ cm^−2^ corresponding to zero (blue curve) and non-zero electric fields (red curve), respectively. Such a fit (dashed lines) quantitatively captures both the linear Stark effect and the stochasticity of the interlayer exciton hybridization. Here, *S*_bkg_ is the fitted reflectance encoding background effects, while *R*_no-TMD_ is the measured reflectance at an optical spot away from the bilayer (Supplementary Section IX). **e**,**f**, The evolution of ${{\mathcal{W}}}_{0}$ and *σ* with the electron density *n* at *T* ≈ 8 K (**e**) and temperature *T* at *n* ≈ 1.3 × 10^12^ cm^−2^ (**f**). We find that both $| {{\mathcal{W}}}_{0}|$ and *σ* increase (decrease) with increasing *n* (*T*), indicating a stronger hybridization between excitons at higher electron densities and lower temperatures. The dashed lines in **e** and **f** represent mean-field trends for the stochastic variance *σ* (Supplementary Section X). The error bars represent combined experimental and fitting uncertainties, as detailed in Supplementary Section IX. Source data

Figure 2a depicts a simulated absorption map ${\rm{Im}\;}[\,\chi(\omega)]$, where *χ*(*ω*) is the polarization response function of the sample (Supplementary Section VII). This simulation corresponds to a simple crossing with ${\mathcal{W}}=0$ and closely resembles the measured signal for *n* = 0 in Fig. 1c. For ${\mathcal{W}}\ne 0$, an avoided crossing occurs, characterized by an asymmetry in intensities between the upper and lower exciton branches (Fig. 2b). This effect is attributed to constructive and destructive interference in the photon emission process of the corresponding exciton branches (Supplementary Section VIII).

While we observe a slight asymmetry in intensities in Fig. 1e, the overall elongated shape at high doping is clearly not captured by either conventional level crossing (Fig. 2a) or anti-crossing (Fig. 2b). Instead, we find that the experimental data are well represented by a model that incorporates ensemble averaging over the coupling ${\mathcal{W}}$, treated as a random, static variable distributed as

2 $$\langle {\mathcal{W}}\rangle ={{\mathcal{W}}}_{0},\qquad \updelta {\mathcal{W}}=({\mathcal{W}}-{{\mathcal{W}}}_{0})\in [-\sigma ,\sigma ].$$

Here, ${{\mathcal{W}}}_{0}$ is the mean coupling strength, while *σ* encodes the variance. The corresponding simulated absorption map (Supplementary Section VII), shown in Fig. 2c, qualitatively agrees with Fig. 1e, capturing two distinctive features: (1) a near-equal intensity distribution between the upper and lower interlayer exciton branches and (2) a plateau-like flattening of the signal along *E*_*z*_ = 0. For this reason, the elongated shape in Fig. 1e is further referred to as stochastic anti-crossing. We emphasize the importance of the static character of the random coupling ${\mathcal{W}}$. If ${\mathcal{W}}$ were instead a time-dependent Markovian variable, its effects would be fully accounted for through a modification of the decay rates *γ*_T_ and *γ*_B_ (Supplementary Section VII).

Using the model in equations (1) and (2), we analyse the experimental data obtained under a variety of different conditions including different temperatures and dopings. Specifically, we simultaneously fit the full reflectance maps (Fig. 1e) with a few-parameter model (Supplementary Section IX), which incorporates substrate reflectance effects and characterizes the interlayer excitons via six parameters: ${{\mathcal{W}}}_{0}$, *σ*, *γ* = *γ*_T_ = *γ*_B_, *d* = *d*_T_ = *d*_B_, *d*_*z*_ and *ω*_0_, which is the bare interlayer exciton energy at *E*_*z*_ = 0. The density and temperature behaviour obtained from this analysis, shown in Fig. 2e,f, reveals that the static stochastic variance *σ* increases with increasing *n* and decreases with increasing *T*. The data also point at the development of a non-zero mean coupling ${{\mathcal{W}}}_{0}\ne 0$ (Supplementary Section XIII), which is consistently found to be relatively small $| {{\mathcal{W}}}_{0}| \ll \sigma$. The mean coupling ${{\mathcal{W}}}_{0}$ roughly follows the trend of *σ*, but for *n* ≈ 1.3 × 10^12^ cm^−2^ vanishes at *T* ≈ 40 K, while *σ* persists up to *T* ≈ 75 K (Supplementary Section IV).

To gain further insights into the nature of this exciton hybridization, we examine both the valley and spin structure of indirect excitons, illustrated in Fig. 1b. With two inequivalent valleys, associated with the *K* and ${K}^{{\prime} }$ points of the hexagonal Brillouin zone (BZ), there are four relevant, optically bright interlayer excitons in total: two excitons with opposite out-of-plane dipoles per valley. The 2*H*-stacked MoS_2_ homobilayer exhibits ${{\mathcal{C}}}_{3}$-rotational symmetry, assigning azimuthal quantum numbers (AQNs) to each of its electronic bands (Fig. 1b). The AQNs of the valence bands are zero, allowing holes to tunnel between layers. Conversely, the AQNs of the conduction bands in the same valley are opposite, which is the fundamental reason that prevents electron tunnelling^9,12,13^ and, thus, naively should prevent interlayer exciton hybridization. The AQNs also dictate the optical selection rules for excitons^9,14,15^: an electron with AQN +1 (−1) corresponds to an exciton coupling to *σ*^+^-polarized (*σ*^−^-polarized) light.

**Fig. 3:**
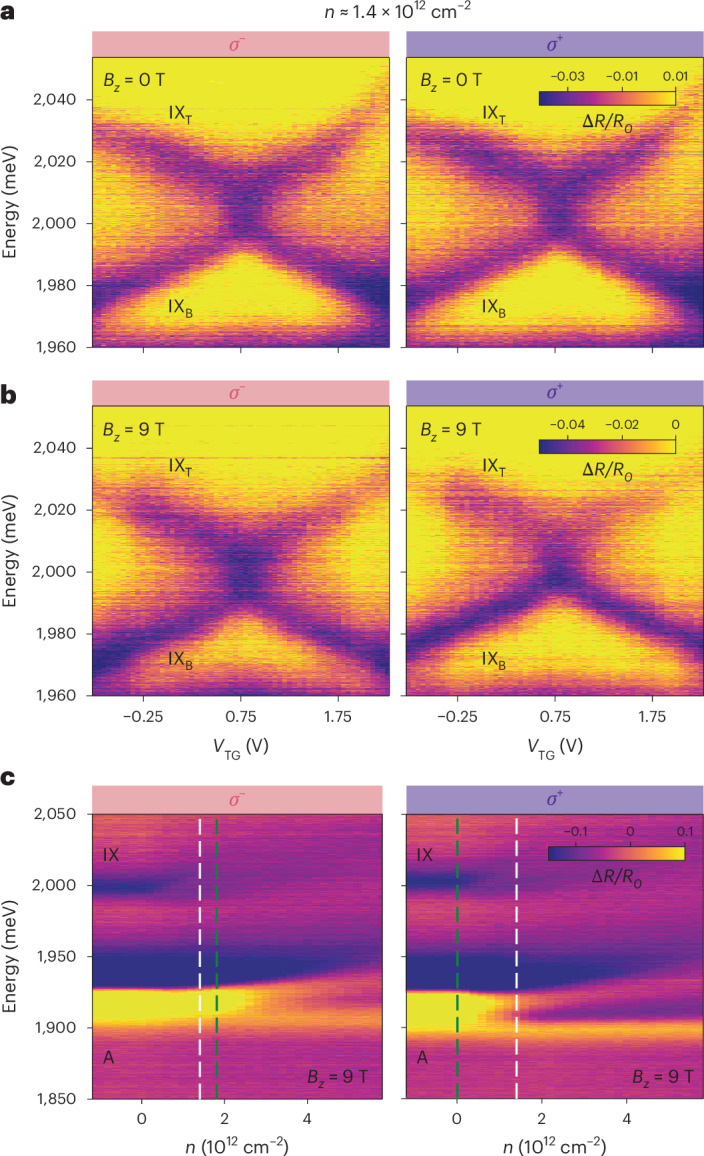
Magnetic field and polarization-resolved properties. **a**,**b**, Electric-field sweeps at *n* ≈ 1.4 × 10^12^ cm^−2^ illustrate the similar appearance of the stochastic anti-crossing for both light polarizations and for both *B*_*z*_ = 0 T (**a**) and *B*_*z*_ = 9 T (**b**). At *B*_*z*_ = 9 T, the *σ*^+^-measurements reveal a small intensity asymmetry between the lower and upper exciton branches, suggesting a slight development of ${{\mathcal{W}}}_{0}$ for this light polarization. **c**, At *B*_*z*_ = 9 T and *n* ≈ 1.4 × 10^12^ cm^−2^ (dashed white lines), conduction-band electrons are expected to be fully spin-polarized. This is supported by density sweeps at *E*_*z*_ = 0 of the *A*-exciton, where the onset of the AP branch for *σ*^−^-polarized light (primarily probing spin-*↑* electrons) is delayed compared with the *σ*^+^-polarized one (essentially sensitive to spin-*↓* electrons); these onsets are indicated with dashed green lines. Source data

One notable feature of MoS_2_ homobilayers is their small conduction-band spin–orbit splitting of a few millielectronvolts, which could result in spin polarization, but not necessarily valley polarization, of conduction-band electrons via an out-of-plane magnetic field *B*_*z*_. This expectation is corroborated by our measurements of polarization-resolved reflection contrast spectra of the intralayer *A*-exciton at *B*_*z*_ = 9 T, *E*_*z*_ = 0 and varying *n* (Fig. 3c). We observe that the attractive polaron (AP) branch for *σ*^−^-polarized light, predominantly sensing spin-*↑* electrons, emerges at a higher doping level compared with the *σ*^+^-polarized one, which primarily probes spin-*↓* electrons^5^. As a result, for electron densities in the asymmetry region between the two AP onsets (Fig. 3c, dashed green lines), conduction-band electrons become fully spin-polarized. In addition, a previous magnetism study on monolayer MoS_2_ (ref. ^5^) suggests that these spin-polarized electrons remain valley-depolarized. For one such representative density *n* ≈ 1.4 × 10^12^ cm^−2^ (Fig. 3c, dashed white lines), we find that the stochastic anti-crossing is robustly present for both light polarizations and for both *B*_*z*_ = 0 (Fig. 3a) and *B*_*z*_ = 9 T (Fig. 3b). Within the error margin of our analysis (Supplementary Section IX), the stochastic variance *σ* is found to be around 15 meV across all four panels in Fig. 3a,b, while the mean coupling ${{\mathcal{W}}}_{0}$ is nearly zero throughout. A slight departure from this trend is that ${{\mathcal{W}}}_{0}$ develops by at most −2 meV for *σ*^+^-polarized light at *B*_*z*_ = 9 T, as indicated by a small intensity asymmetry between the upper and lower exciton branches in Fig. 3b (right). The persistent presence of a large *σ* nearly independent of *B*_*z*_ indicates that the interlayer exciton hybridization is predominantly agnostic to the electron spin.

We now turn to the theoretical interpretation of our observations. The stochastic anti-crossing in Fig. 1e can be attributed to intravalley and/or intervalley interlayer exciton hybridization. The modest asymmetry in the lower and upper branches (associated with a small non-zero mean coupling ${{\mathcal{W}}}_{0}\ne 0$ in the model given by equations (1) and (2)) is probably due to the intervalley scenario, as interlayer excitons within any of the two valleys have opposite AQNs and the optical interference effects that give rise to ${{\mathcal{W}}}_{0}\ne 0$ are suppressed for excitons with opposite polarizations (Supplementary Section VIII). By contrast, the stochastic variance *σ* ≠ 0 is compatible with both scenarios (Supplementary Section VIII), suggesting that both types of hybridization can play a role.

Hybridization between intervalley interlayer excitons with opposing dipoles is allowed from a symmetry perspective, as these excitons, such as ${{\rm{IX}}}_{{\rm{T}},{{\rm{K}}}^{{\prime} }}$ and IX_B,K_ (depicted in red in Fig. 1b), have the same AQNs. Even without electron doping the sample, these could hybridize with each other via direct Coulomb interactions: either via exciton exchange^16,17^, expected to be weak because of the reduced transition dipole moment of interlayer excitons compared with intralayer ones, or via a process involving the scattering of the ${{\rm{IX}}}_{{\rm{T}},{{\rm{K}}}^{{\prime} }}$-exciton electron and hole across the TMD BZ, which is suppressed because it occurs at a large momentum ${\boldsymbol{K}}-{{\boldsymbol{K}}}^{{\prime} }$ and involves electron and hole layer switching (Supplementary Section XI). Thus, such direct coupling is expected to be weak, consistent with $| {{\mathcal{W}}}_{0}| \lesssim 2\,{\rm{meV}}$ for *n* = 0 (Fig. 2e). Doping the sample could enhance such hybridization mechanisms via simple effects such as trion formation, polaronic dressing or Fermi sea fluctuations, possibly explaining the emergence of non-zero mean coupling ${{\mathcal{W}}}_{0}\ne 0$ and the density trend in Fig. 2e (such dynamical electron-enhanced exciton hybridization is still expected to be suppressed, consistent with our measurements in Fig. 2e,f, as further discussed in Supplementary Section XI). The intensity asymmetry in Fig. 3b (right) could arise from the presence of doped electrons indistinguishable from the corresponding exciton electron. Increasing temperature weakens polaronic dressing effects^18^ and increases exciton scattering off phonons^19^, which reduces exciton wave-function overlaps. Both effects may contribute to explaining decreasing $| {{\mathcal{W}}}_{0}|$ with increasing *T* as observed in Fig. 2f.

At the same time, the emergence of the stochastic variance *σ* involves quasi-static processes, which are beyond the simple dynamical processes mentioned previously, especially given the large values of *σ* in Fig. 2e,f. Moreover, the effects of quenched disorder or charge traps should be mitigated via electron screening, particularly because strongly interacting regimes in TMDs can be achieved at substantially higher electron densities than in conventional semiconductors^4,7,20^. Experimentally, we observed *σ* increases as *n* increases, which invalidates disorder-induced scenarios. Furthermore, the inversion symmetry of the sample suggests that the system is unlikely to be ferroelectric (a conclusion supported experimentally in Supplementary Section IV), and the absence of amplitude doubling in the stochastic anti-crossing (Fig. 1f) indicates that nonlinearities in *E*_*z*_ are probably not relevant.

Instead, *σ* could originate from a correlated many-body state that develops an order parameter *Δ*, in which case the observed stochastic behaviour is attributed to quasi-static spatial fluctuations of this order parameter. In particular, one potential candidate is interlayer electron coherence, corresponding to an exchange instability akin to the typical emergence of ferromagnetism. This correlated state has been proposed theoretically^21^ and experimentally established in quantum Hall bilayers^22–29^, where the strong magnetic field quenches the electron kinetic energy and, thus, favours an ordered phase, but it has not yet been conclusively observed at *B*_*z*_ = 0. Such a state requires (1) strong Coulomb interactions $1\ll{r}_{{\mathrm{s}}}\equiv {m}^{\ast}{{{e}}}^{2}/(4\uppi{\varepsilon}_{0}\varepsilon {\hslash}^{2}\sqrt{\uppi\,n})$ (${m}^{\ast}$ and *ε* are the effective electron mass and permittivity of the surrounding medium, respectively), (2) the absence of electron tunnelling and (3) a small interlayer separation *lk*_F_ ≪ 1 (*k*_F_ is the Fermi momentum and *l* ≈ 0.6 nm is the interlayer separation).

Our experimental conditions in the studied MoS_2_ homobilayer naturally fulfil these stringent prerequisites. First, the large effective mass ${m}^{\ast}$ ≈ 0.7*m*_e_ and the small permittivity of hexagonal boron nitride (hBN), *ε* ≈ 3.76 (ref. ^30^), result in *r*_s_ ≈ 20 for *n* = 1 × 10^12^ cm^−2^ and *r*_s_ ≈ 11.5 for *n* = 3 × 10^12^ cm^−2^. Second, due to the intravalley conduction-band AQN mismatch in Fig. 1b and as experimentally confirmed in ref. ^13^, electron tunnelling between the layers is intrinsically absent. Third, we estimate *lk*_F_ ≈ 0.2 for *n* = 3 × 10^12^ cm^−2^. Finally, by studying samples with varying hBN thickness to modulate the strength of Coulomb interactions, we confirm the Coulomb origin of the studied phenomenon (Supplementary Section III).

**Fig. 4:**
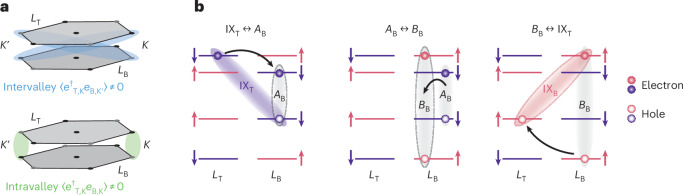
Coulomb-mediated mechanism of interlayer exciton hybridization. **a**, The electronic many-body state can exhibit interlayer electron coherence with intervalley (top) or intravalley (bottom) correlations. **b**, Intravalley coherence leads to an effective order-parameter-induced electron tunnelling-like process (left), resulting in hybridization between IX_T_- and *A*_B_-excitons (shown is the *K* valley). The *A*_B_-exciton couples to the IX_B_ state via exciton exchange (middle) followed by hole tunnelling (right), thereby hybridizing the two interlayer excitons.

The putative emergence of interlayer electron coherence may manifest as the stochastic anti-crossing via a Coulomb-mediated mechanism in Fig. 4b, consistent with and potentially explaining our observations. In conventional semiconductor double-quantum wells, the order parameter is associated with the spontaneous breaking of U(1) symmetry, corresponding to in-plane rotations of the layer pseudospin; the up and down directions of the pseudospin represent the top and bottom layers, respectively (for simplicity, we omit discussion of electron spin). In MoS_2_ homobilayers, the presence of two valleys enriches this symmetry to U(1) × SU(2), where the SU(2) part is related to valley pseudospin rotations (Supplementary Section XI discusses the approximate nature of this U(1) × SU(2) symmetry in TMDs). This enlarged symmetry places intervalley (Fig. 4a, top) and intravalley (Fig. 4a, bottom) correlations on equal footing (Supplementary Section X). The significance of intravalley correlations, such as $$| \varDelta | {e}^{i\varphi } \approx$$$$\langle {e}_{{\rm{T}}}^{\dagger }{e}_{{\rm{B}}}\rangle \ne 0$$ (Fig. 4a, bottom), where *e*_B_ and $${e}_{{\rm{T}}}^{\dagger }$$ are the *K*-valley electron annihilation and creation operators, respectively, is that they lead to strong Coulomb-mediated electron tunnelling-like processes^23,31–35^ (Supplementary Section X). In momentum space, these can be expressed as follows (we write only the processes in the *K* valley):

3 $$-\sum _{{\mathbf{k}}}{t}_{{\mathbf{k}}}{\hat{e}}_{{\rm{B}},{\rm{K}}}^{\dagger }({\mathbf{k}}){\hat{e}}_{{\rm{T,K}}}({\mathbf{k}})+\,\text{h.c.}\,,$$

where the coupling constant *t*_**k**_, which provides an effective tunnelling-like rate, is determined by both the order parameter amplitude ∣*Δ*(**r**)∣ and phase *φ*(**r**). Assuming perfect Hartree–Fock correlations^21^ and taking into account the ångström-scale interlayer separation between the TMD layers, we estimate *t*_**k**_ ≈ 100 meV for *n* = 2 × 10^12^ cm^−2^ (Supplementary Section X). Although this estimate is crude, it underscores the significance of the proposed processes.

This electron tunnelling-like process gives rise to a hybridization of, for example, IX_T_- and *A*_B_-excitons (Fig. 4b, left), with the corresponding coupling estimated to be of the order ${t}_{{{\rm{IX}}}_{{\rm{T}}}\leftrightarrow {{{A}}}_{{\rm{B}}}}\approx 85\,{\rm{meV}}$ for *n* = 2 × 10^12^ cm^−2^ (Supplementary Section X). The *A*_B_-exciton is, in turn, coupled to the IX_B_ state via a two-step process shown in Fig. 4b (middle and right), involving exciton exchange^36^ (Fig. 4b, middle) followed by hole tunnelling^9,10^ (Fig. 4b, right). This *A*_B_–IX_B_ coupling is already established experimentally^37^, and its strength is estimated to be about ${t}_{{A}_{{\rm{B}}}\leftrightarrow {{\rm{IX}}}_{{\rm{B}}}}\approx 4\,{\rm{meV}}$. Combined, the processes in Fig. 4b result in the hybridization of the two interlayer excitons IX_T_ and IX_B_, with the coupling strength being of the order of 5 meV for *n* = 2 × 10^12^ cm^−2^ (Supplementary Section X). Although the above analysis relies on two simplifying assumptions—perfect Hartree–Fock correlations and a perturbative approach to relating the electronic order parameter to interlayer exciton hybridization—the estimated number is comparable to the measured values in Fig. 2e. Finally, the exciton exchange step in Fig. 4b (middle) involves flipping both exciton electron and hole spins, indicating that the proposed mechanism is relevant even when conduction-band electrons are spin-polarized by a magnetic field (Fig. 3), provided the system remains valley-depolarized^5^.

The corresponding interlayer exciton hybridization $\updelta {\mathcal{W}}({\mathbf{r}})$ is determined by the interlayer electron coherence ∣*Δ*(**r**)∣*e*^*iφ*(**r**)^ and, thus, inherits its spatial inhomogeneities arising from statistical fluctuations of the order parameter phase *φ*(**r**). Typically, these fluctuations take the form of vortices; however, in TMDs with the enlarged U(1) × SU(2) symmetry, other meron-like topological defects might be essential^38^. In our experiment, the coupling $\updelta {\mathcal{W}}({\mathbf{r}})$ is spatially averaged over the optical spot size of about 0.5 μm. This size is expected to be much larger than the phase coherence length (at low temperatures, it is on the order of the correlation length of the disorder potential^39^, which we expect to be at most a few hundred nanometres). As a result, upon spatial averaging, the order parameter induced contribution to the interlayer exciton hybridization vanishes $\langle \updelta {\mathcal{W}}({\mathbf{r}})\rangle =0$ (see also Supplementary Section V, where we experimentally explore optical size effects and argue against the phase coherence as the origin of the mean coupling ${{\mathcal{W}}}_{0}$ in equation (2)). Nevertheless, an appreciable stochastic variance *σ* in equation (2) can develop because it is essentially determined by the order parameter amplitude ∣*Δ*(**r**)∣. The observed behaviour in Fig. 2e,f for *σ* is consistent with the development of the amplitude ∣*Δ*∣ as electron density *n* increases within the range accessible in our experiment (*r*_s_ ≈ 10–20), and its gradual suppression with increasing temperature *T* until eventual melting; both these trends are well captured by the mean-field analysis, as indicated by the dashed lines in Fig. 2e,f (Supplementary Section X). Although our study reaches a maximum electron density *n* of about 3 × 10^12^ cm^−2^, further increases in *n* (decreases in *r*_s_) should eventually melt the electron coherence^21^, an expectation supported by the absence of electron tunnelling observed at *r*_s_ ≈ 3 (ref. ^13^).

Our observations open up exciting opportunities for exploring strongly correlated many-body phenomena in bilayer systems, particularly in understanding magnetic exchange instabilities—one of the important challenges in modern condensed matter physics. Experimentally, the challenge lies in controllably entering and probing a strongly interacting regime, while theoretically, the phase diagram for *r*_s_ ≈ 10–20 (as in our experiment), where the electronic system is between a simple Fermi liquid and crystalline states^7,20^, is not yet fully understood, with only limited Monte Carlo data. In this context, MoS_2_ homobilayers offer a key advantage as we can naturally access this strongly interacting regime, while interlayer excitons represent a unique optical probe of pseudospin correlations.

Our observations have close connections with several fundamental many-body phenomena expected in bilayer systems, such as interlayer exciton condensation^28,40,41^ and interlayer electron superconductivity^42^. We remark that a small twist between the TMD layers breaks the ${{\mathcal{C}}}_{3}$-rotational symmetry and gives rise to a small direct electron tunnelling. This tunnelling is expected to stabilize the order parameter phase coherence and lead to more coherent rather than stochastic hybridization between interlayer excitons. In addition, the application of an in-plane magnetic field might enable the exploration of the Pokrovsky–Talapov phase transition^43^ (Supplementary Section XI discusses that, even without twisting, electron pair tunnelling events can occur, but their role is yet to be fully understood). Furthermore, the TMD valley degree of freedom is expected to enrich the phase diagram compared with conventional semiconductors, as the order parameter is likely to have multiple components (Fig. 4a and Supplementary Section X); understanding the structure of spatial order parameter inhomogeneities and their interplay with disorder warrants further theoretical investigation.

Another exciting avenue for future research is to explore the coherence properties of strongly interacting indirect excitons. Our work demonstrates that these can be substantially influenced by tuning the many-body electron system, potentially enabling novel quantum optics applications. We envision that, similar to the interlayer exciton coupling observed here, electron doping of MoS_2_ trilayers might lead to the hybridization of quadrupolar excitons^10,44^, which could have promising applications for sensing in the terahertz domain and quantum information processing^45^. Further insight into exciton interactions could potentially be gained through time-resolved spectroscopy techniques.

## Methods

### Device fabrication

2*H*-stacked bilayer MoS_2_, hBN and few-layer-graphite were exfoliated from bulk crystals onto silicon substrates with a 285-nm silicon oxide layer. Bilayer MoS_2_ flakes were identified according to the reflectance contrast under an optical microscope. The thickness of the hBN flakes was measured by an atomic force microscope. Four graphite/hBN/BL MoS_2_/hBN/graphite heterostructures were fabricated using the dry transfer method^46^, where electrical contacts were made to the MoS_2_ and the graphite gates using 10-nm Cr and 100-nm Au deposited via electron beam evaporation. Data from device 1, with top/bottom hBN thicknesses of 19 nm/24 nm, are presented in the main text. Devices 2 and 3 are fabricated with top/bottom hBN thicknesses of 36 nm/38 nm and 32 nm/16 nm, respectively. Device 4 uses thin hBN layers as gate dielectrics, where the top/bottom hBN is 5.4 nm/6.3 nm thick.

### Optical spectroscopy

Polarization-resolved measurements were conducted in a Bluefors dilution refrigerator. All other optical measurements were carried out in a Montana Instruments cryostat (base temperature *T* = 8 K), using a custom-built 4f confocal set-up with a Zeiss objective (100×, numerical aperture 0.75, working distance 4 mm). Reflectance spectra were measured using a halogen source (Thorlabs SLS201L) and a spectrometer (Acton SpectroPro 2300i). Electrostatic gating was performed with Keithley 2400 sourcemeters.

## Online content

Any methods, additional references, Nature Portfolio reporting summaries, source data, extended data, supplementary information, acknowledgements, peer review information; details of author contributions and competing interests; and statements of data and code availability are available at 10.1038/s41567-025-02971-0.

## Supplementary information

Supplementary information Supplementary Figs. 1–16 and discussion.

## Source data

Source Data Fig. 1 Unprocessed source data.

Source Data Fig. 2 Unprocessed source data.

Source Data Fig. 3 Unprocessed source data.

## Data Availability

Source data are provided with this paper. All other data supporting the findings of this study are available from the corresponding author upon reasonable request.

## References

[1] He, K. et al. Tightly bound excitons in monolayer WSe_2_. *Phys. Rev. Lett.* **113**, 026803 (2014). 25062219 10.1103/PhysRevLett.113.026803

[2] Sidler, M. et al. Fermi polaron-polaritons in charge-tunable atomically thin semiconductors. *Nat. Phys.* **13**, 255–261 (2017).

[3] Xu, Y. et al. Correlated insulating states at fractional fillings of moiré superlattices. *Nature* **587**, 214–218 (2020). 33177668 10.1038/s41586-020-2868-6

[4] Smoleński, T. et al. Signatures of Wigner crystal of electrons in a monolayer semiconductor. *Nature* **595**, 53–57 (2021). 34194018 10.1038/s41586-021-03590-4

[5] Roch, J. G. et al. Spin-polarized electrons in monolayer MoS_2_. *Nat. Nanotechnol.* **14**, 432–436 (2019). 30858519 10.1038/s41565-019-0397-y

[6] Ciorciaro, L. et al. Kinetic magnetism in triangular moiré materials. *Nature* **623**, 509–513 (2023). 37968525 10.1038/s41586-023-06633-0 PMC10651480

[7] Sung, J. et al. An electronic microemulsion phase emerging from a quantum crystal-to-liquid transition. *Nat. Phys.* **21**, 437–443 (2025).

[8] Jiang, Y., Chen, S., Zheng, W., Zheng, B. & Pan, A. Interlayer exciton formation, relaxation, and transport in TMD van der Waals heterostructures. *Light Sci. Appl.* **10**, 72 (2021). 33811214 10.1038/s41377-021-00500-1 PMC8018964

[9] Gerber, I. C. et al. Interlayer excitons in bilayer MoS_2_ with strong oscillator strength up to room temperature. *Phys. Rev. B* **99**, 035443 (2019).

[10] Leisgang, N. et al. Giant Stark splitting of an exciton in bilayer MoS_2_. *Nat. Nanotechnol.* **15**, 901–907 (2020). 32778806 10.1038/s41565-020-0750-1

[11] Deilmann, T. & Thygesen, K. S. Interlayer excitons with large optical amplitudes in layered van der Waals materials. *Nano Lett.* **18**, 2984–2989 (2018). 29665688 10.1021/acs.nanolett.8b00438

[12] Gong, Z. et al. Magnetoelectric effects and valley-controlled spin quantum gates in transition metal dichalcogenide bilayers. *Nat. Commun.* **4**, 2053 (2013). 23784147 10.1038/ncomms3053

[13] Pisoni, R. et al. Absence of interlayer tunnel coupling of *K*-valley electrons in bilayer MoS_2_. *Phys. Rev. Lett.* **123**, 117702 (2019). 31573263 10.1103/PhysRevLett.123.117702

[14] Cao, T. et al. Valley-selective circular dichroism of monolayer molybdenum disulphide. *Nat. Commun.* **3**, 887 (2012). 22673914 10.1038/ncomms1882 PMC3621397

[15] Xiao, D., Liu, G.-B., Feng, W., Xu, X. & Yao, W. Coupled spin and valley physics in monolayers of MoS_2_ and other group-VI dichalcogenides. *Phys. Rev. Lett.* **108**, 196802 (2012). 23003071 10.1103/PhysRevLett.108.196802

[16] Pikus, G. E. & Bir, G. L. Exchange interaction in excitons in semiconductors. *J. Exp. Theor. Phys.* **33**, 108 (1971).

[17] Yu, H., Liu, G.-B., Gong, P., Xu, X. & Yao, W. Dirac cones and Dirac saddle points of bright excitons in monolayer transition metal dichalcogenides. *Nat. Commun.* **5**, 3876 (2014). 24821438 10.1038/ncomms4876

[18] Mulkerin, B. C., Tiene, A., Marchetti, F. M., Parish, M. M. & Levinsen, J. Exact quantum virial expansion for the optical response of doped two-dimensional semiconductors. *Phys. Rev. Lett.* **131**, 106901 (2023). 37739378 10.1103/PhysRevLett.131.106901

[19] Selig, M. et al. Excitonic linewidth and coherence lifetime in monolayer transition metal dichalcogenides. *Nat. Commun.* **7**, 13279 (2016). 27819288 10.1038/ncomms13279 PMC5103057

[20] Zhou, Y. et al. Bilayer Wigner crystals in a transition metal dichalcogenide heterostructure. *Nature* **595**, 48–52 (2021). 34194017 10.1038/s41586-021-03560-w

[21] Zheng, L., Ortalano, M. & Sarma, S. D. Exchange instabilities in semiconductor double-quantum-well systems. *Phys. Rev. B* **55**, 4506–4515 (1997).

[22] Sarma, S. D. & Pinczuk, A. *Perspectives in Quantum Hall Effects: Novel Quantum Liquids in Low-Dimensional Semiconductor Structures* (John Wiley & Sons, 2008).

[23] Spielman, I., Eisenstein, J., Pfeiffer, L. & West, K. Resonantly enhanced tunneling in a double layer quantum Hall ferromagnet. *Phys. Rev. Lett.* **84**, 5808–5811 (2000). 10991060 10.1103/PhysRevLett.84.5808

[24] Kellogg, M., Eisenstein, J., Pfeiffer, L. & West, K. Vanishing hall resistance at high magnetic field in a double layer two-dimensional electron system. *Phys. Rev. Lett.* **93**, 036801 (2004). 15323851 10.1103/PhysRevLett.93.036801

[25] Kellogg, M., Spielman, I., Eisenstein, J., Pfeiffer, L. & West, K. Observation of quantized Hall drag in a strongly correlated bilayer electron system. *Phys. Rev. Lett.* **88**, 126804 (2002). 11909491 10.1103/PhysRevLett.88.126804

[26] Spielman, I., Eisenstein, J., Pfeiffer, L. & West, K. Observation of a linearly dispersing collective mode in a quantum Hall ferromagnet. *Phys. Rev. Lett.* **87**, 036803 (2001). 11461580 10.1103/PhysRevLett.87.036803

[27] Fertig, H. Energy spectrum of a layered system in a strong magnetic field. *Phys. Rev. B* **40**, 1087–1095 (1989). 10.1103/physrevb.40.1087 9991931

[28] Shi, Q. et al. Bilayer WSe_2_ as a natural platform for interlayer exciton condensates in the strong coupling limit. *Nat. Nanotechnol.* **17**, 577–582 (2022). 35437321 10.1038/s41565-022-01104-5

[29] Fertig, H. & Murthy, G. Coherence network in the quantum hall bilayer. *Phys. Rev. Lett.* **95**, 156802 (2005). 16241749 10.1103/PhysRevLett.95.156802

[30] Laturia, A., Van de Put, M. L. & Vandenberghe, W. G. Dielectric properties of hexagonal boron nitride and transition metal dichalcogenides: from monolayer to bulk. *NPJ 2D Mater. Appl.* **2**, 6 (2018).

[31] Lin, K. A. et al. Emergence of interlayer coherence in twist-controlled graphene double layers. *Phys. Rev. Lett.* **129**, 187701 (2022). 36374684 10.1103/PhysRevLett.129.187701

[32] Wen, X.-G. & Zee, A. Tunneling in double-layered quantum Hall systems. *Phys. Rev. B* **47**, 2265–2270 (1993). 10.1103/physrevb.47.2265 10006266

[33] Stern, A., Sarma, S. D., Fisher, M. P. & Girvin, S. Dissipationless transport in low-density bilayer systems. *Phys. Rev. Lett.* **84**, 139–142 (2000). 11015854 10.1103/PhysRevLett.84.139

[34] Stern, A., Girvin, S. M., MacDonald, A. H. & Ma, N. Theory of interlayer tunneling in bilayer quantum Hall ferromagnets. *Phys. Rev. Lett.* **86**, 1829–1832 (2001). 11290259 10.1103/PhysRevLett.86.1829

[35] Fogler, M. M. & Wilczek, F. Josephson effect without superconductivity: realization in quantum Hall bilayers. *Phys. Rev. Lett.* **86**, 1833–1836 (2001). 11290260 10.1103/PhysRevLett.86.1833

[36] Guo, L. et al. Exchange-driven intravalley mixing of excitons in monolayer transition metal dichalcogenides. *Nat. Phys.* **15**, 228–232 (2019).

[37] Sponfeldner, L. et al. Capacitively and inductively coupled excitons in bilayer MoS_2_. *Phys. Rev. Lett.* **129**, 107401 (2022). 36112433 10.1103/PhysRevLett.129.107401

[38] Girvin, S. M. & Yang, K. *Modern Condensed Matter Physics* (Cambridge Univ. Press, 2019).

[39] Rossi, E., Núnez, A. S. & MacDonald, A. Interlayer transport in bilayer quantum Hall systems. *Phys. Rev. Lett.* **95**, 266804 (2005). 16486385 10.1103/PhysRevLett.95.266804

[40] Wang, Z. et al. Evidence of high-temperature exciton condensation in two-dimensional atomic double layers. *Nature* **574**, 76–80 (2019). 31578483 10.1038/s41586-019-1591-7

[41] Ma, L. et al. Strongly correlated excitonic insulator in atomic double layers. *Nature* **598**, 585–589 (2021). 34707306 10.1038/s41586-021-03947-9

[42] Zhao, D. et al. Evidence of finite-momentum pairing in a centrosymmetric bilayer. *Nat. Phys.* **19**, 1599–1604 (2023).

[43] Yang, K. et al. Spontaneous interlayer coherence in double-layer quantum Hall systems: symmetry-breaking interactions, in-plane fields, and phase solitons. *Phys. Rev. B* **54**, 11644–11658 (1996). 10.1103/physrevb.54.11644 9984954

[44] Yu, L. et al. Observation of quadrupolar and dipolar excitons in a semiconductor heterotrilayer. *Nat. Mater.* **22**, 1485–1491 (2023). 37857888 10.1038/s41563-023-01678-y

[45] Yelin, S. & Hemmer, P. Resonantly enhanced nonlinear optics in semiconductor quantum wells: an application to sensitive infrared detection. *Phys. Rev. A* **66**, 013803 (2002).

[46] Kim, K. et al. Van der Waals heterostructures with high accuracy rotational alignment. *Nano Lett.* **16**, 1989–1995 (2016). 26859527 10.1021/acs.nanolett.5b05263

