# Supplementary material for: Optical signatures of interlayer electron coherence in a bilayer semiconductor
Source: Nat Phys. 2025 Aug 20;21(10):1563–9. doi: 10.1038/s41567-025-02971-0 (PMC12518136; doi:10.1038/s41567-025-02971-0)
Supplement: Supplementary file 1 — Supplementary Figs. 1–16 and discussion. [file 41567_2025_2971_MOESM1_ESM.pdf]

nature physics

Supplementary information https://doi.org/10.1038/s41567-025-02971-0

Optical signatures of interlayer electron coherence in a bilayer semiconductor

In the format provided by the authors and unedited

CONTENTS

I. Device electrostatics 2
A. Dual-gated voltage scan 2
B. Electrostatic model 3

II. Reproducibility of the observations 4

III. Evidence for the Coulomb origin of the stochastic anti-crossing 6

IV. Temperature effects 6

V. Optical spot size effects 7

VI. Evidence for the absence of ferroelectricity 8

VII. Static stochastic avoided crossing 9
A. Modeling the fluctuating coupling 9
B. Analysis of the static fluctuations scenario 9
C. Analysis of the dynamic fluctuations scenario 10

VIII. Development of a nonzero mean coupling $\mathcal{W}_0 \neq 0$ 10
A. Experimental evidence 10
B. Hybridization of interlayer excitons with the same AQNs 11
C. Additional considerations for data analysis 11

IX. Data processing 12
A. Fitting the intrinsic data 13
B. Fitting the doped data 13
C. Error bar analysis 13

X. Theory for the stochastic anti-crossing 14
A. Intravalley interlayer exciton hybridization and crude estimates 15
B. Additional symmetry considerations for MoS_2-homobilayers 16
C. Self-consistent Hartree-Fock analysis 17

XI. Intervalley interlayer exciton hybridization, weak symmetry breaking, and Fermi sea fluctuations 20
A. Anomalous terms and the U(1) layer symmetry 20
B. Second-order perturbation theory 21

References 23

I. DEVICE ELECTROSTATICS

A. Dual-gated voltage scan

Doping the sample modifies the A-exciton intensity and lineshape, splitting its resonance into attractive and repulsive polaron branches. By keeping track of the A-exciton, we, therefore, can decipher the doping behavior of the MoS_2-homobilayer (Fig. S1).

A representative differential reflectivity spectrum and a sweep as a function of doping (along the zero electric-field line) of device 1 are shown in Fig. S1A,C, where we identify the A-, IX-, and B-excitons. We represent the oscillator strength of the A-exciton as the integrated area under the positive part of the respective Fano-resonance peak (shaded grey; red dashed lines denote the boundaries of the integrated energy range). Initially, the A-exciton intensity remains fairly constant. As electrons are doped into the MoS_2, the A-exciton intensity decreases, indicated by the blue dashed line in Fig. S1C,D. Integrating over the area at each (V_{TG}, V_{BG})-pair and normalizing by the maximum integrated area, we obtain the 2D map in Fig. S1B. The brighter/darker region represents a higher/lower A-exciton oscillator strength and, thus, a lower/higher doping level. Due to the Fermi-level pinning to the MoS_2 conduction band, only electron doping is achievable in our applied gate-voltage range [1–4]. The direction of the electric-field sweeps described in the main text is illustrated in the 2D map in Fig. S1B and Fig. S2B as the white dashed line. The black dashed line is the predicted $E_z = 0$ line from our electrostatic simulations in Sec. I B (Fig. S2). While our simulations might not be quantitatively accurate due to simplified assumptions about the real system, the electric-field sweeps conducted in the main text always cross the $E_z = 0$ line. Since we have not observed degeneracy of $\mathrm{IX_T}$- and $\mathrm{IX_B}$-excitons at any

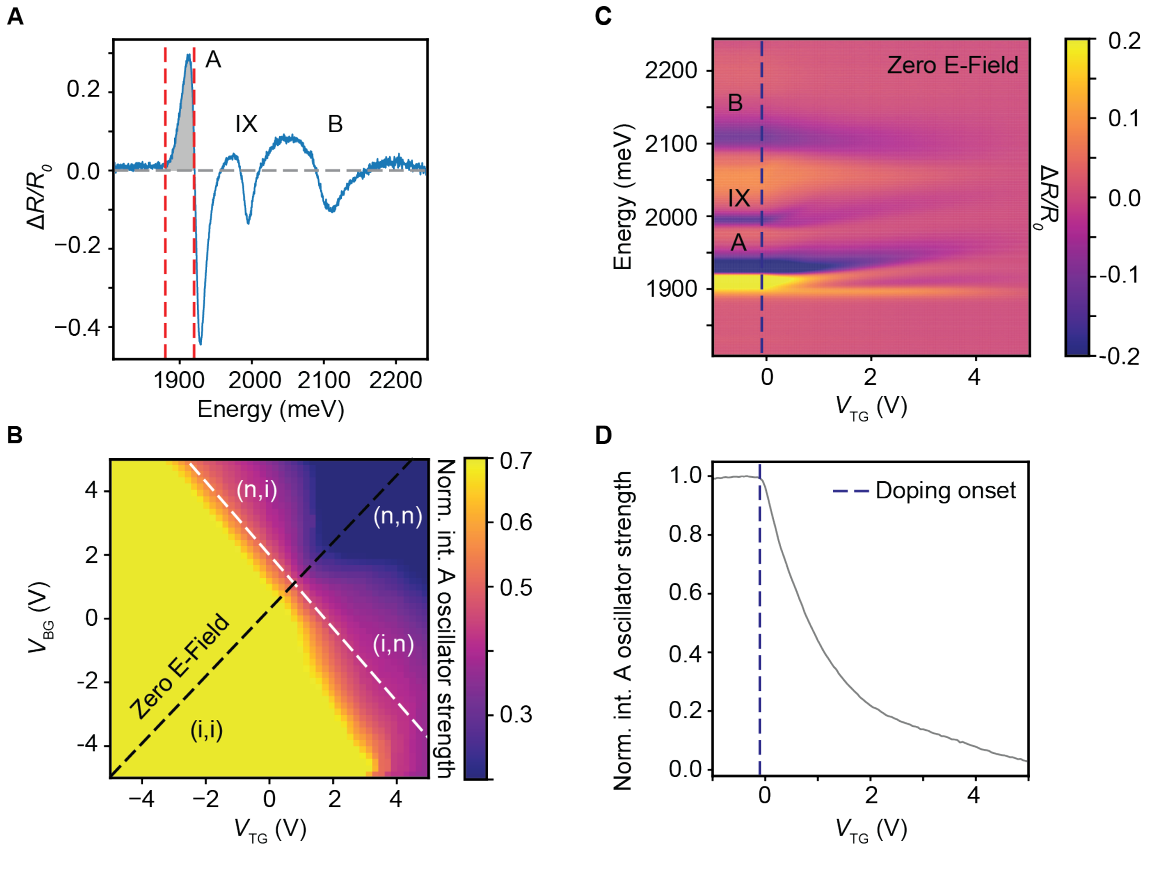

FIG. S1. Optical response as a function of gate voltage. **A** Representative differential reflectivity spectrum $\Delta R/R_0$ of the MoS_2-homobilayer (device 1). The A-exciton oscillator strength is represented by integrating the area under the positive part of the A-exciton Fano-resonance, indicated by the grey shaded area. **B** Dual-gated voltage map of the extracted A-exciton oscillator strength. Both MoS_2 layers are intrinsic (i,i) in the yellow region and electron-doped (n,n) in the blue region. (i,n) and (n,i) label the purple regions where one layer is intrinsic while the other is doped. The black dashed line denotes the simulated $E_z = 0$ line. **C** Voltage-sweep along the $E_z = 0$ line encoding the evolution of A-, IX-, B-excitons with doping. **D** A-exciton oscillator strength extracted from **C**. The blue dashed line indicates the doping onset.

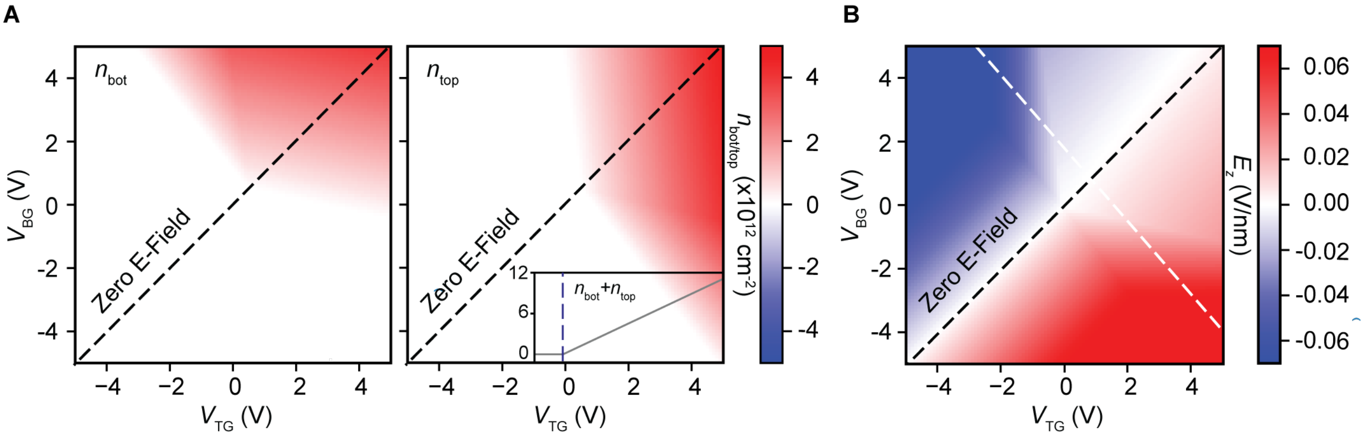

FIG. S2. Simulated electrostatics. **A** Simulated doping level in the bottom and top MoS_2 layers (device 1), respectively. The inset plots the total intensity along the zero electric-field line. The blue dashed line indicates the doping onset as extracted from the measurements. **B** Dual-gated voltage map of the simulated electric field E_z. The white dashed line encodes the direction of the electric-field sweeps performed in the main text.

gate voltage along such sweeps and for a finite density $n \neq 0$, we conclude that these excitons become non-degenerate at $E_z = 0$ when the sample is doped.

B. Electrostatic model

We model the electrostatic properties of the sample as follows. Simple capacitance equations – relating the electric potentials at the top gate $V_{\rm TG}$, bottom gate $V_{\rm BG}$, top TMD layer $\phi_{\rm top}$, and bottom TMD layer $\phi_{\rm bot}$ to the corresponding carrier densities $n_{\rm T}$, $n_{\rm B}$, $n_{\rm top}$, and $n_{\rm bot}$ – are given by:

$$en_{\rm T} = \frac{\epsilon_{\rm hBN}}{d_{\rm T}}(V_{\rm TG} - \phi_{\rm top}), \tag{S1a}$$

$$en_{\rm top} = \frac{\epsilon_{\rm TMD}}{d_{\rm TMD}}(\phi_{\rm top} - \phi_{\rm bot}) - \frac{\epsilon_{\rm hBN}}{d_{\rm T}}(V_{\rm TG} - \phi_{\rm top}), \tag{S1b}$$

$$en_{\rm bot} = \frac{\epsilon_{\rm hBN}}{d_{\rm B}}(\phi_{\rm bot} - V_{\rm BG}) - \frac{\epsilon_{\rm TMD}}{d_{\rm TMD}}(\phi_{\rm top} - \phi_{\rm bot}), \tag{S1c}$$

$$en_{\rm B} = -\frac{\epsilon_{\rm hBN}}{d_{\rm B}}(\phi_{\rm bot} - V_{\rm BG}). \tag{S1d}$$

Here, $\epsilon_{\rm hBN} = 3.76$ and $\epsilon_{\rm TMD} = 6.4$ are the permittivities of hBN and MoS_2; $d_{\rm T}$, $d_{\rm B}$, and $d_{\rm TMD} = 0.65$ nm are the top hBN, bottom hBN, and bilayer MoS_2 thicknesses, respectively. Equations (S1) are consitent with the charge neutrality condition $n_{\rm T} + n_{\rm top} + n_{\rm bot} + n_{\rm B} = 0$. The TMD sample is grounded and in electro-chemical equilibrium with the corresponding contact, resulting in the conditions:

$$\phi_{\rm top} + \frac{\mu_{\rm top}}{e} = 0, \tag{S2a}$$

$$\phi_{\rm bot} + \frac{\mu_{\rm bot}}{e} = 0, \tag{S2b}$$

where $\mu_{\rm top}$ and $\mu_{\rm bot}$ label the chemical potentials of the top and bottom MoS_2 layers, respectively. Disregarding the possibility of hole doping, neglecting the small conduction-band spin-orbit splitting, and assuming the electron state is well captured via a simple Fermi liquid, we relate the TMD densities $n_{\rm top}$, $n_{\rm bot}$ to the corresponding chemical potentials $\mu_{\rm top}$ and $\mu_{\rm bot}$ via

$$n_{\rm top} = \frac{2m_{\rm e}^* k_{\rm B}T}{\pi\hbar^2}\ln\Big(1 + e^{(\mu_{\rm top}-\mu_0)/k_{\rm B}T}\Big), \tag{S3a}$$

$$n_{\rm bot} = \frac{2m_{\rm e}^* k_{\rm B}T}{\pi\hbar^2}\ln\Big(1 + e^{(\mu_{\rm bot}-\mu_0)/k_{\rm B}T}\Big), \tag{S3b}$$

where $m_e^* = 0.454m_e$ is the effective electron mass and μ_0 is a fitting parameter chosen to ensure that the simulated density onset matches our measurements – see Fig. S1C,D. We numerically solve this set of equations for n_{top} and n_{bot} and calculate the total carrier density $n = n_{top} + n_{bot}$ as stated in the text.

II. REPRODUCIBILITY OF THE OBSERVATIONS

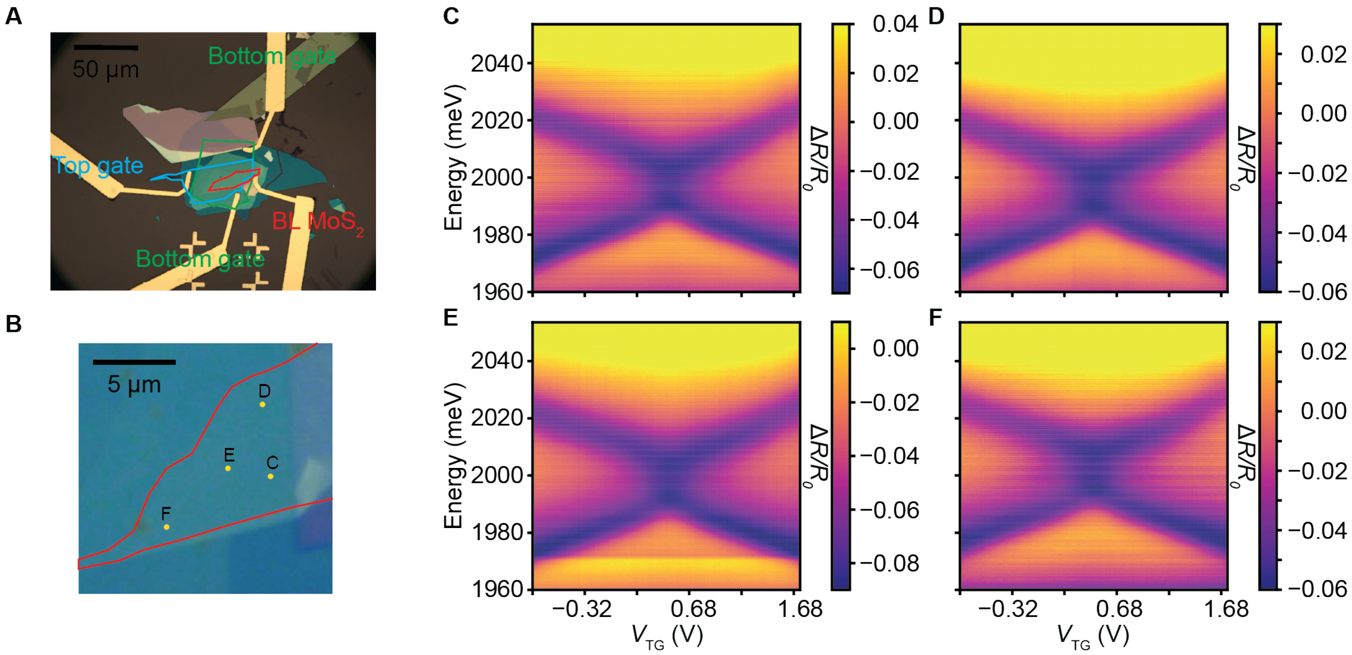

FIG. S3. Reproducibility of the stochastic anti-crossing within the same device. **A**-**B** Microscope images of the main device 1; the red line marks the dual-gated MoS_2-homobilayer region. **C**-**F** Electric-field sweeps at four representative optical spots marked in **A** confirming that the stochastic anti-crossing is robustly present across the sample.

Reproducibility within the same device.—Figure S3 shows that the stochastic anti-crossing is robustly present across the entire spatial extend of the main device (device 1), confirming that this effect is highly reproducible within the same sample.

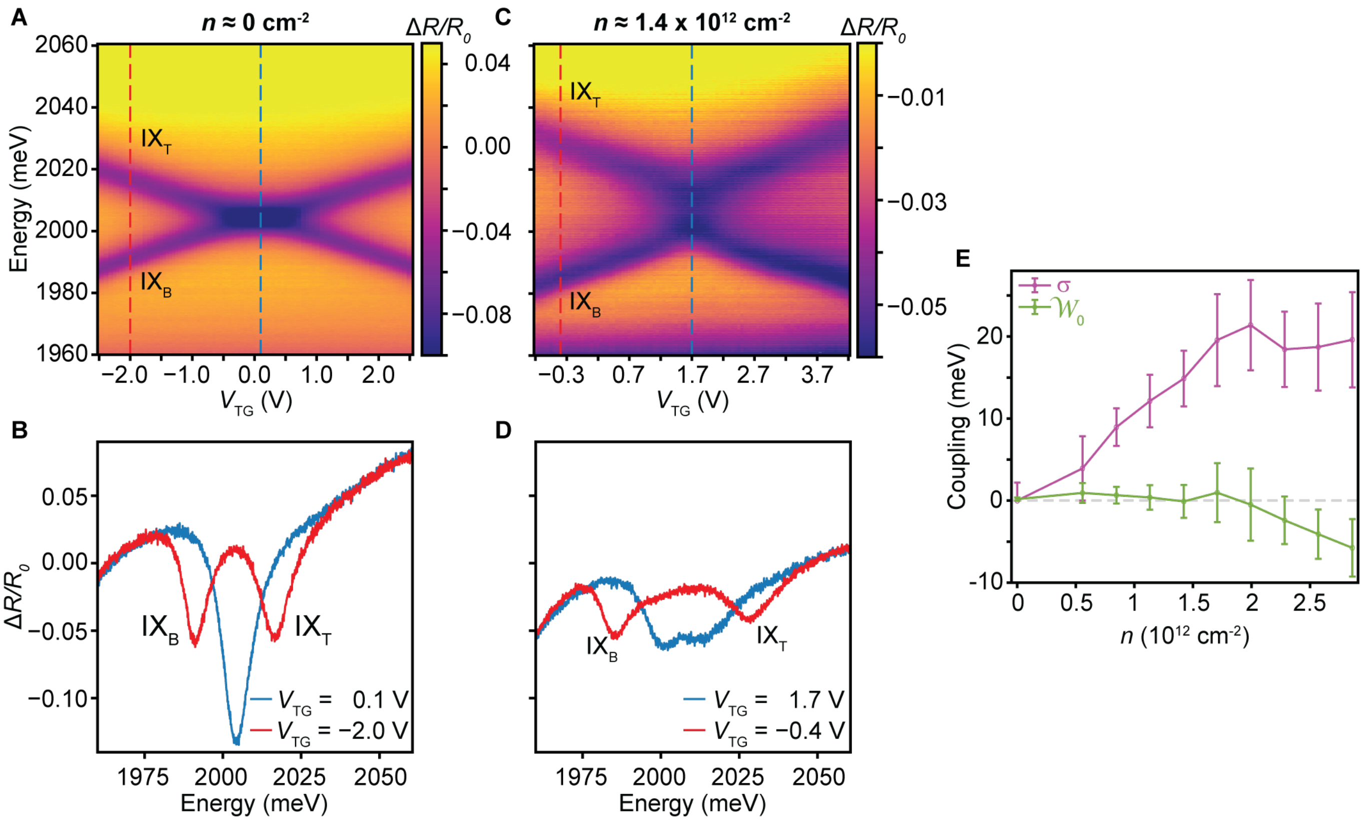

FIG. S4. The stochastic anti-crossing in device 2. **A** Electric-field sweep in the intrinsic region ($n \approx 0$), demonstrating a simple interlayer exciton crossing. **B** Two linecuts at $E_z = 0$ and $E_z \neq 0$, marked by white dashed lines in **A**, illustrate amplitude doubling at the degeneracy point $E_z = 0$. **C,D** Similar to **A,B**, but for the doped system, revealing a transition from a simple crossing to a stochastic crossing. In the doped case, the linecut at $E_z = 0$ no longer exhibits amplitude doubling. **E** Evolution of the mean coupling $\mathcal{W}_0$ and the variance σ with electron density, obtained by fitting the 2D reflectance maps (see Sec. IX below), showing quantitative agreement with the results presented in Fig. 2 of the main text. Error bars represent combined experimental and fitting uncertainties (see Sec. IX below).

Reproducibility in other devices.—We fabricated two additional devices (device 2 and device 3) with roughly (but not exactly) the same geometry. Figure S4 shows the stochastic anti-crossing measurements in device 2 (device 3 is not shown as a similar effect was observed there), confirming not only the reproducibility of this phenomenon in another device but also the robustness of our data analysis. The extracted values for $\mathcal{W}_0$ and σ agree quantitatively with those for device 1 in Fig. 2 of the main text.

III. EVIDENCE FOR THE COULOMB ORIGIN OF THE STOCHASTIC ANTI-CROSSING

To investigate the Coulomb origin of the stochastic anti-crossing, we fabricated another device (designated as device 4) using thin hBNs as gate dielectrics, as described in the Methods section. This new device features a substantially different surrounding Coulomb environment compared to devices 1-3 with thick hBNs. Additionally, the thin hBN dielectric enables screening of Coulomb interactions in the MoS_2-homobilayer from the graphite gates due to the short distance between the sample and the gates [5]. Figure S5 demonstrates that device 4 no longer exhibits the stochastic anti-crossing, as upon doping the sample the exciton spectral weight at $E_z = 0$ remains roughly twice that of the individual excitons. The absence of stochastic anti-crossing is attributed to the mentioned screening effect and confirms the Coulomb origin of the observed phenomenon.

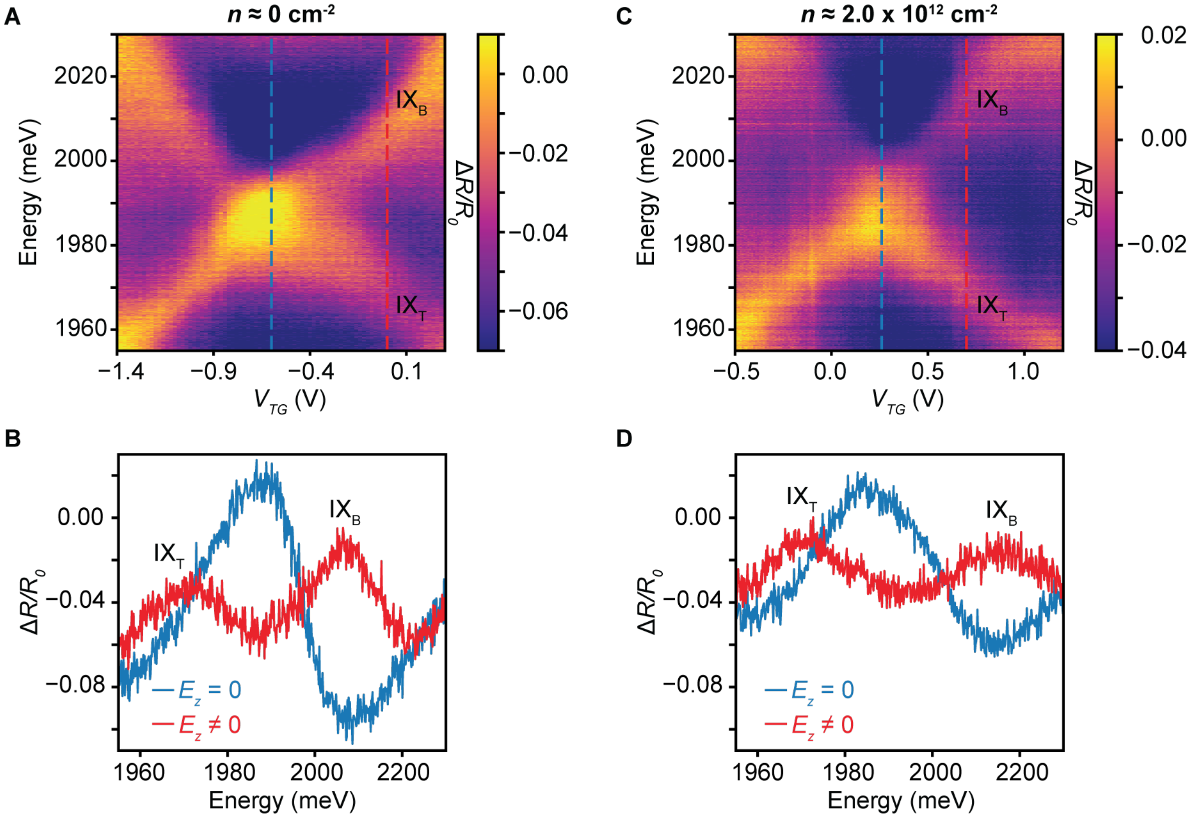

FIG. S5. Coulomb origin of the stochastic anti-crossing. **A** Electric-field sweeps for the undoped case in device 4, with thin hBNs as gate dielectrics, show the crossing of two interlayer excitons at the degeneracy point $E_z = 0$ ($T = 4\,\mathrm{K}$). Linecuts (white dashed lines in **A**) at $E_z = 0$ and $E_z \neq 0$ in **B** reveal amplitude doubling at $E_z = 0$. In device 4, interlayer excitons exhibit peaks, rather than dips, in reflectance spectra, attributed to interference with the thin-hBN background reflectivity. **C,D** Doping this thin-hBN sample does not disrupt the crossing and degeneracy of the two interlayer excitons at $E_z = 0$.

IV. TEMPERATURE EFFECTS

Figure S6 presents raw data from stochastic anti-crossing measurements at $n \approx 1.3 \times 10^{12}\,\mathrm{cm}^{-2}$, comparing low ($T = 7.9\,\mathrm{K}$) and high ($T = 75\,\mathrm{K}$) temperatures. At $T = 7.9\,\mathrm{K}$, stochastic hybridization is evident, while at $T = 75\,\mathrm{K}$, it transitions to a simple crossing, characterized by amplitude doubling at the degeneracy point $E_z = 0$ in Fig. S6D. While at higher temperatures processes such as exciton scattering off phonons become progressively more important and result in excitonic line broadening, this broadening alone does not fully account for the observed relative amplitude change. For this reason, and following the main text, we interpret our temperature-dependent measurements as evidence of order parameter melting, see also Sec. X.

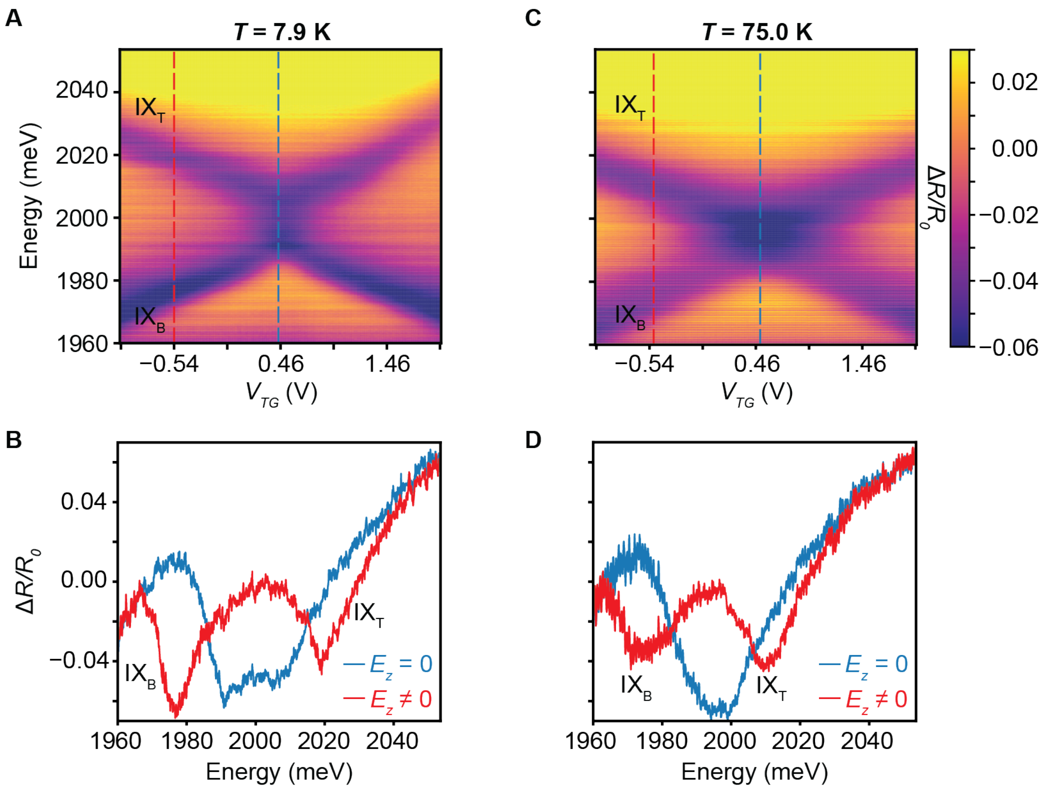

FIG. S6. Electric-field sweeps at two representative temperatures at $n \approx 1.3 \times 10^{12}\,\mathrm{cm}^{-2}$. The stochastic DC Stack effect, clearly observed at $T = 7.9\,\mathrm{K}$ (panels **A** and **B**), disappears at $T = 75.0\,\mathrm{K}$ (panels **C** and **D**) and turns into a simple crossing, as further evidenced by the amplitude doubling at the degeneracy point $E_z = 0$ in **D** (see also Fig. S4B).

V. OPTICAL SPOT SIZE EFFECTS

In the main text, we proposed that the static stochastic variance σ originates from an order parameter – in the form of interlayer electron coherence – of the many-body electron system. We attribute the origin of this static stochasticity σ to immobile spatial order parameter fluctuations. In principle, if the phase coherence length ξ is comparable to the optical spot size R, this order parameter could also contribute to the mean coupling $\mathcal{W}_0$. Furthermore, it is tempting to attribute the observed trends of $\mathcal{W}_0$ in Fig. 2E,F to this long-range scenario. We remark that if the optical spot size can be made small, such that $\xi \gtrsim R$, the interlayer exciton hybridization should be coherent without strong static stochastic features, implying that we expect $\xi \lesssim R$ in the experiment.

One way to test this long-range scenario is to study how the stochastic anti-crossing varies with the optical spot size, as we expect that the order-parameter contribution to the mean coupling behaves as $\delta\mathcal{W}_0 \propto \xi/R$. Figure S7A,B shows that upon increasing the optical spot size by about an order of magnitude, extracted by fitting the camera image of a laser spot with a 2D Gaussian, both the stochastic variance σ and the mean coupling $\mathcal{W}_0$ roughly remain intact. These data are, therefore, consistent with the picture where the phase coherence length is appreciably smaller than the smallest optical spot size of about $0.6\,\mu\mathrm{m}$, so that the order parameter can contribute to σ but not to $\mathcal{W}_0$.

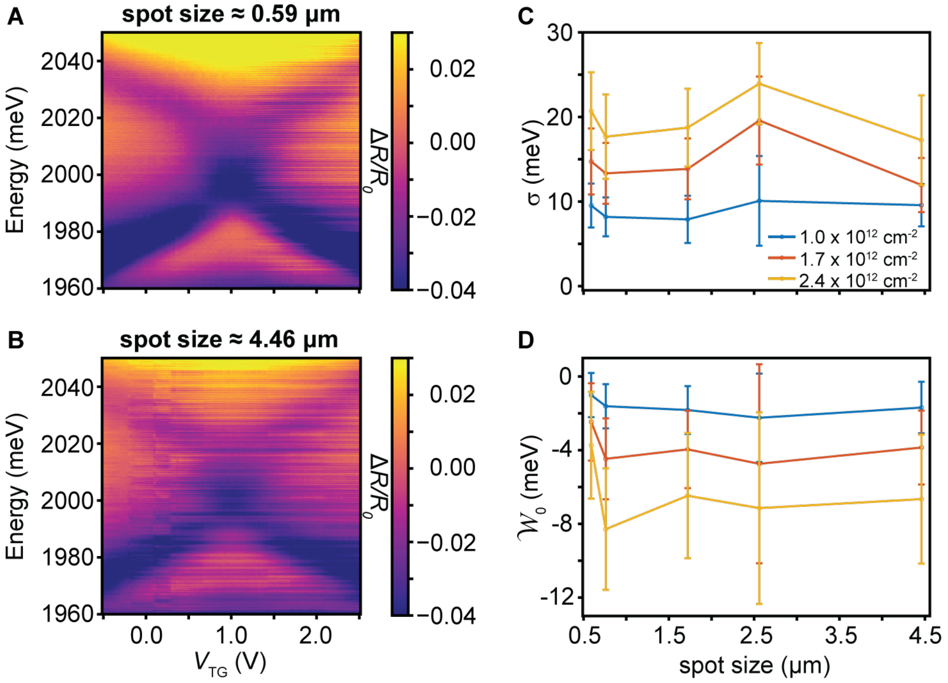

FIG. S7. Optical spot size effects. **A** and **B** show electric-field sweeps at $n \approx 2.33 \times 10^{12}\,\mathrm{cm}^{-2}$ for a focused (**A**) and defocused (**B**) optical spot size. **C** and **D** depict fitted values of σ and $\mathcal{W}_0$, respectively, which do not exhibit a clear dependence on the optical spot size. Error bars represent combined experimental and fitting uncertainties (see Sec. IX below).

VI. EVIDENCE FOR THE ABSENCE OF FERROELECTRICITY

Ferroelectric materials exhibit spontaneous electric polarization that can be reversed by an applied electric field, leading to characteristic hysteresis in their response. This behavior is typically observed as a dependence of polarization on the history of the applied field, resulting in different states for forward and backward voltage sweeps.

In our experiments, we probe the presence of ferroelectricity by performing voltage sweeps in both forward and backward directions at finite doping. As shown in Fig. S8, the optical spectra remain identical for both sweep directions, indicating a fully reversible response. This suggests that no ferroelectric polarization is retained under our experimental conditions, ruling out the presence of robust ferroelectric order in the system.

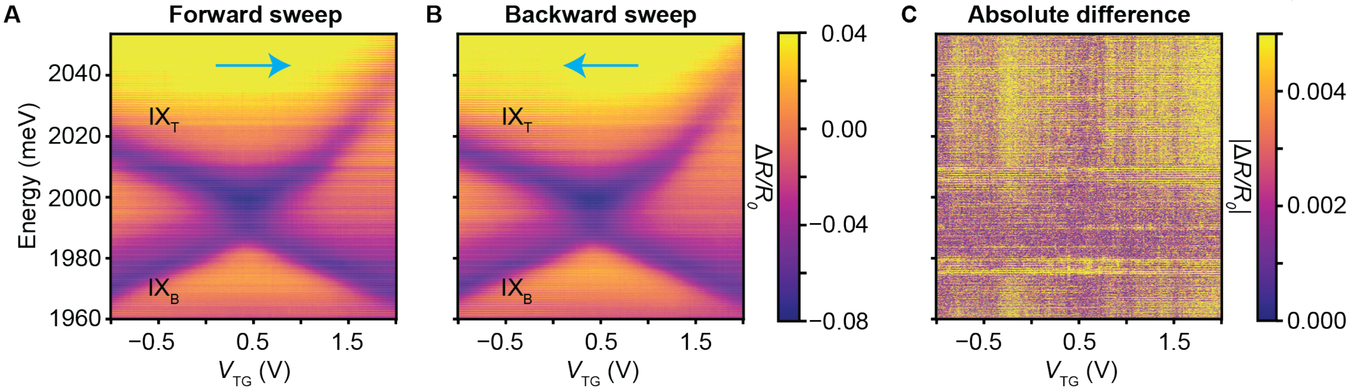

FIG. S8. Voltage sweep tuning the electric field at finite doping in **A** forward and **B** backward direction, indicated by the blue arrows. The spectra are identical for both sweep directions. **(C)** Absolute difference between the optical responses for forward and backward gate sweeps, showing no hysteresis effect. This indicates that no ferroelectric polarization is retained in the system under our experimental conditions.

VII. STATIC STOCHASTIC AVOIDED CROSSING

In this section, we detail our phenomenological modeling of the stochastic crossing representing our measurements of the DC Stark effect at a finite electron density.

The two interlayer excitons with opposite out-of-plane dipole moments can be modeled by a simple model of two coupled harmonic oscillators (here we set the zero energy to be the interlayer exciton energy at the degeneracy point $E_z = 0$):

$$i\partial_t X_{\rm T} = \omega_{\rm T} X_{\rm T} - i\gamma_{\rm T} X_{\rm T} + \mathcal{W} X_{\rm B} - d_{\rm T}\mathcal{E}_d(t), \quad i\partial_t X_{\rm B} = \omega_{\rm B} X_{\rm B} - i\gamma_{\rm B} X_{\rm B} + \mathcal{W} X_{\rm T} - d_{\rm B}\mathcal{E}_d(t). \tag{S4}$$

Here $\omega_{\rm T/B} = \pm d_z E_z$ encodes the bare excitonic energies that linearly shift with the out-of-plane electric field E_z; d_z is the out-of-plane dipole moment; $\gamma_{\rm T/B}$ is the total linewidth of the corresponding excitonic resonance; $\mathcal{W}$ is the coupling strength between the two excitonic branches; $d_{\rm T/B}$ is the transition dipole moment (assumed to be real in our modeling) encoding the response to the probe field $\mathcal{E}_d$. The observable we are interested in is the imaginary part of the susceptibility $\text{Im}[\chi(\omega)]$ defined as $\mathcal{P}(\omega) = \chi(\omega)\mathcal{E}_d(\omega)$, where $\mathcal{P}(\omega) = d_{\rm T} X_{\rm T}(\omega) + d_{\rm B} X_{\rm B}(\omega)$ is the TMD excitonic polarization.

A. Modeling the fluctuating coupling

We consider two scenarios for the fluctuating coupling $\mathcal{W}$:

- **Static fluctuations model**: $\mathcal{W}$ is time-independent but the measured signal represents an average over the distribution with

$$\langle \mathcal{W} \rangle = \mathcal{W}_0, \qquad \delta\mathcal{W} = (\mathcal{W} - \mathcal{W}_0) \in [-\sigma, \sigma]. \tag{S5}$$

- **Dynamic white-noise averaging**:

$$\langle \mathcal{W}(t) \rangle = \mathcal{W}_0, \qquad \langle \delta\mathcal{W}(t)\delta\mathcal{W}(t') \rangle = \gamma\delta(t - t'). \tag{S6}$$

We note that in the former limit, the coupling $\mathcal{W}(t)$ can, in principle, be time-dependent, but its dynamics should occur on timescales much longer than the exciton dynamics, set by the splitting $\Delta = 2d_z E_z$ as well as by the decay rates $\gamma_{\rm T}$ and $\gamma_{\rm B}$. The latter scenario corresponds to the opposite limit where the dynamics of $\mathcal{W}(t)$ are much faster than any other relevant timescales, allowing for the approximation of the variable $\mathcal{W}(t)$ as Markovian.

B. Analysis of the static fluctuations scenario

For a given $\mathcal{W}$, the response function is given by:

$$\chi_{\mathcal{W}}(\omega) = \frac{-1}{(\omega - \Delta/2 + i\gamma_{\rm T})(\omega + \Delta/2 + i\gamma_{\rm B}) - \mathcal{W}^2} \begin{bmatrix} d_{\rm T} \\ d_{\rm B} \end{bmatrix}^{\rm T} \begin{bmatrix} \omega + \Delta/2 + i\gamma_{\rm B} & \mathcal{W} \\ \mathcal{W} & \omega - \Delta/2 + i\gamma_{\rm T} \end{bmatrix} \begin{bmatrix} d_{\rm T} \\ d_{\rm B} \end{bmatrix}. \tag{S7}$$

We are interested in the average response $\langle \chi_{\mathcal{W}}(\omega) \rangle$ over the distribution in Eq. (S5). This computation can be done analytically, and it boils down to evaluating the following integrals:

$$I_1 = \frac{1}{2C}\int_{-\sigma}^{\sigma} \frac{d\mathcal{W}}{2\sigma}\left[\frac{1}{\mathcal{W} + \mathcal{W}_0 - C} - \frac{1}{\mathcal{W} + \mathcal{W}_0 + C}\right], \quad I_2 = \frac{1}{2}\int_{-\sigma}^{\sigma} \frac{d\mathcal{W}}{2\sigma}\left[\frac{1}{\mathcal{W} + \mathcal{W}_0 - C} + \frac{1}{\mathcal{W} + \mathcal{W}_0 + C}\right], \tag{S8}$$

where $C^2 = (\omega - \Delta/2 + i\gamma_{\rm T})(\omega + \Delta/2 + i\gamma_{\rm B})$. These integrals can be computed using $\int_{-\sigma}^{\sigma} \frac{d\mathcal{W}}{\mathcal{W} + A} = \log[A + \sigma] - \log[A - \sigma]$. We verified that numerical averaging of Eq. (S7) matches the results obtained from the analytical approach. The resulting response function is shown in Fig. S9 and well represents the measured data. We remark that we use here the uniform distribution in Eq. (S5) only for numerical convenience, and any other reasonable distribution (e.g., Gaussian) could be used instead.

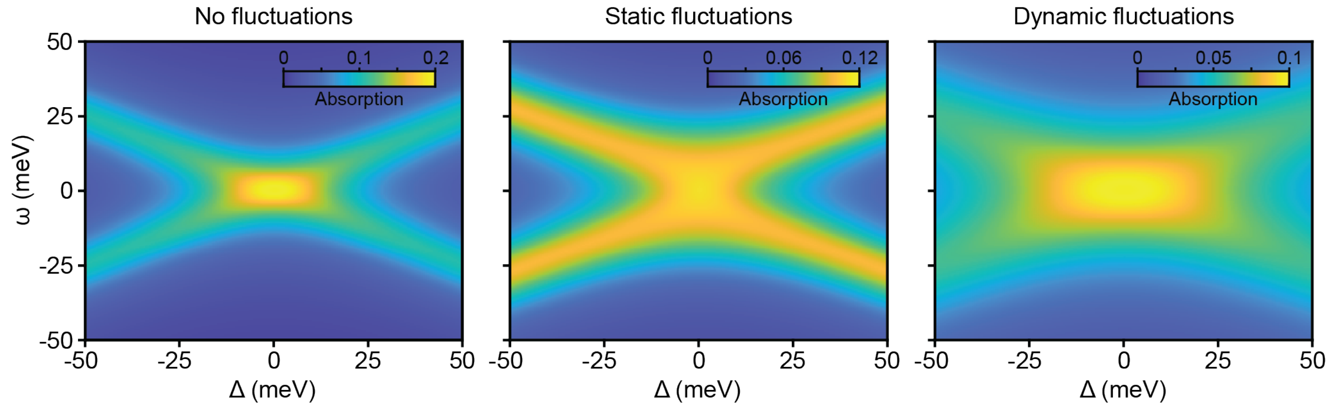

FIG. S9. Absorption maps Im[χ] for the two scenarios of fluctuating coupling. The left panel depicts the case of uncoupled interlayer excitons. The middle panel illustrates the static stochastic scenario with $\mathcal{W}_0 = 0$ and $\sigma = 20\,\mathrm{meV}$. The right panel shows the dynamic scenario with $\gamma = 20\,\mathrm{meV}$. Parameters used: $d_\mathrm{T} = d_\mathrm{B}$, $\gamma_\mathrm{T} = \gamma_\mathrm{B} = 10\,\mathrm{meV}$.

C. Analysis of the dynamic fluctuations scenario

Dynamic modeling requires additional considerations as the fluctuating coupling term, $\delta\mathcal{W}(t)$, introduces multiplicative noise. Therefore, it may be essential to consider parametric processes. To see this point explicitly, we write the equations of motion in the frequency domain:

$$\hat{\mathcal{G}}_{0,\omega}\begin{bmatrix}X_\mathrm{T}(\omega)\\X_\mathrm{B}(\omega)\end{bmatrix} - \int_{-\infty}^{\infty}\frac{d\omega'}{2\pi}\begin{bmatrix}0 & \delta\mathcal{W}_{\omega-\omega'}\\ \delta\mathcal{W}_{\omega-\omega'} & 0\end{bmatrix}\begin{bmatrix}X_\mathrm{T}(\omega')\\X_\mathrm{B}(\omega')\end{bmatrix} = -\mathcal{E}_d(\omega)\begin{bmatrix}d_\mathrm{T}\\d_\mathrm{B}\end{bmatrix}, \quad \hat{\mathcal{G}}_{0,\omega} = \begin{bmatrix}\omega-\Delta/2+i\gamma_\mathrm{T} & -\mathcal{W}_0\\ -\mathcal{W}_0 & \omega+\Delta/2+i\gamma_\mathrm{B}\end{bmatrix}.$$

The second term encodes the mentioned frequency mixing due to the dynamics of $\delta\mathcal{W}(t)$, which can cause a parametric instability provided $\delta\mathcal{W}(t)$ contains Fourier harmonics at frequencies commensurate with the bare Rabi oscillations. In our case, we assume a broadband white noise $\langle\delta\mathcal{W}_\omega\delta\mathcal{W}_{\omega'}\rangle = 2\pi\gamma\delta(\omega+\omega')$, which contains all possible frequency harmonics, including the commensurate ones.

There are various ways to approach the problem at hand, including the direct numerical sampling over the dynamical noise, and among them, the most efficient appears to be the non-equilibrium Green's function technique developed in Ref. [6]. If one is interested solely in the retarded susceptibility, the self-consistent Born approximation turns out to be exact so that the solution can be written as:

$$\hat{\Sigma}^R_\omega = -i\frac{\gamma}{2}\hat{1} \Rightarrow \begin{bmatrix}X_\mathrm{T}(\omega)\\X_\mathrm{B}(\omega)\end{bmatrix} = -\mathcal{E}_d(\omega)[\hat{\mathcal{G}}_{0,\omega} - \hat{\Sigma}^R_\omega]^{-1}\begin{bmatrix}d_\mathrm{T}\\d_\mathrm{B}\end{bmatrix}. \tag{S9}$$

In other words, the effects of dynamical fluctuations are fully captured via the substitution:

$$\gamma_\mathrm{T} \to \gamma_\mathrm{T} + \gamma/2, \qquad \gamma_\mathrm{B} \to \gamma_\mathrm{B} + \gamma/2. \tag{S10}$$

The dynamical fluctuations model can, thus, be understood as a microscopic model of a pure dephasing channel. The resulting response function, plotted in Fig. S9 (right panel), clearly does not capture the observations.

VIII. DEVELOPMENT OF A NONZERO MEAN COUPLING $\mathcal{W}_0 \neq 0$

In this section, we present experimental evidence that a nonzero mean coupling $\mathcal{W}_0 \neq 0$ develops at a finite electron density and low temperatures. We also discuss the implications of $\mathcal{W}_0 \neq 0$ for the nature of interlayer exciton hybridization and for rigorous data processing.

A. Experimental evidence

In our modeling of stochastic anti-crossing in Sec. VII, a nonzero coupling $\mathcal{W}_0 \neq 0$ is the only parameter that results in an asymmetry between the upper and lower excitonic branches (see also the discussion below in this section

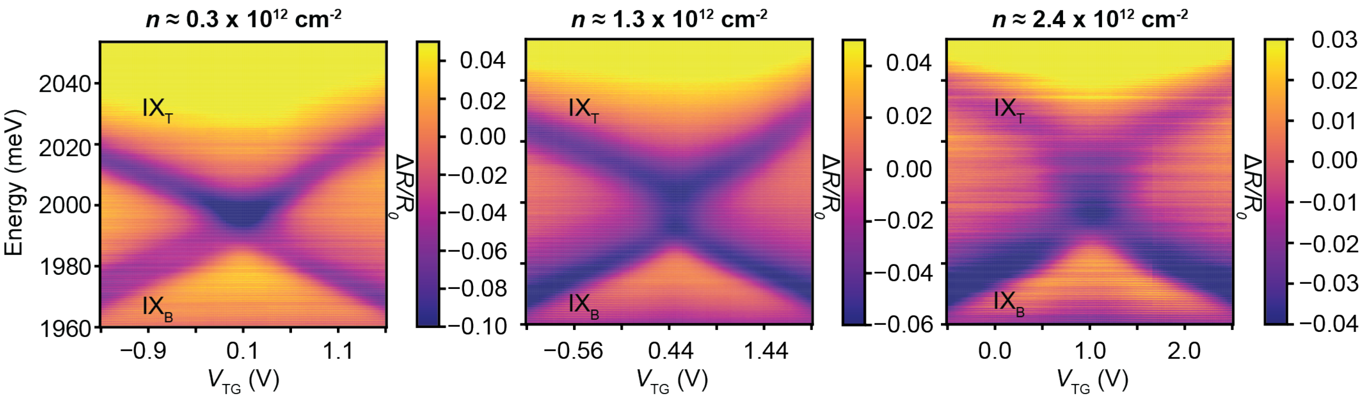

FIG. S10. Electric-field sweeps at three representative carrier densities at $T = 8$ K: $n \approx 0.34 \times 10^{12}\,\mathrm{cm}^{-2}$ (left), $n \approx 1.26 \times 10^{12}\,\mathrm{cm}^{-2}$ (middle), and $n \approx 2.33 \times 10^{12}\,\mathrm{cm}^{-2}$ (right). The evolution from the upper exciton branch initially being stronger than the lower branch, then becoming equal, and eventually becoming weaker, indicates the development of a non-zero $\mathcal{W}_0 \neq 0$.

as well as Sec. IX B), as seen, for instance, in Fig. 2 of the main text. This effect can be understood as the onset of superradiant and subradiant states of the two coupled dipoles.

Figure S10 shows the evolution of the stochastic anti-crossing with electron density: at low dopings (left panel), the upper branch exhibits slightly stronger oscillator strength; at intermediate dopings (middle panel), the strengths of the two exciton branches are comparable; at high dopings (right panel), the lower branch is brighter, while the upper branch weakens and eventually becomes barely observable. As such, the data in Fig. S10 indicate the emergence of exciton hybridization $\mathcal{W}_0 \neq 0$.

B. Hybridization of interlayer excitons with the same AQNs

One immediate implication of a nonzero $\mathcal{W}_0 \neq 0$ is that interlayer excitons with the same AQNs hybridize with each other. This implies that excitons in the opposite valleys hybridize with each other, as excitons within the same valley have opposite AQNs and cannot interfere with each other (i.e., subradiant and superradiant states cannot form in this case). This conclusion is further illustrated by a simple simulation as in Sec. VII – see Fig. S11. Our analysis, however, does not rule out the possibility that excitons within the same valley can also hybridize (Fig. S11), which might be relevant in case $\mathcal{W}_0$ and σ have different origins, as further discussed in the main text and in Secs. X and XI.

C. Additional considerations for data analysis

Below we analyze the experimental data using the static fluctuations model, encoded in Eqs. (S4) and (S5), and here we discuss two additional considerations needed for robust data processing:

- Effects of σ and $\gamma_{\mathrm{T/B}}$: We first note that for $\Delta = 0$, distinguishing the effects of σ from linewidth broadenings γ_{T} and γ_{B} can be challenging. However, when $|\Delta| \gtrsim \sigma$, the excitonic peak linewidths are primarily determined by γ_{T} and γ_{B}. Therefore, in our data analysis, we simultaneously consider the entire 2D reflectivity map to unambiguously determine σ; we also fix $\gamma_{\mathrm{T}} = \gamma_{\mathrm{B}} = \gamma$, where γ is assumed to be independent of the applied electric field.

- Effects of a finite $\mathcal{W}_0 \neq 0$ and $d_{\mathrm{T}} \neq d_{\mathrm{B}}$: We also note that two additional factors can manifest in the measured reflectivity maps as $\mathcal{W}_0 \neq 0$. One of these is interference effects from the background, which we found to be unimportant – see Sec. II and Fig. S4E. The other factor arises from the hybridization between the interlayer excitons with the B-excitons [7], resulting from hole tunneling. The B-excitons favor the higher-energy interlayer exciton to be brighter, an effect that can be captured via $d_{\mathrm{T}} \neq d_{\mathrm{B}}$ but difficult to unambiguously disentangle from $\mathcal{W}_0 \neq 0$. On the other hand, this asymmetry effect due to the B-excitons is expected to be weak, as further supported by $|\mathcal{W}_0| \lesssim 2\,\mathrm{meV}$ for the intrinsic region ($n = 0$). To avoid overfitting, we, thus, set $d_{\mathrm{T}} = d_{\mathrm{B}} = d$, where d is assumed to be electric field independent. This assumption is intuitive because the two interlayer excitons should be degenerate at $\Delta = 0$, but it can lead to a small systematic error in determining $\mathcal{W}_0$.

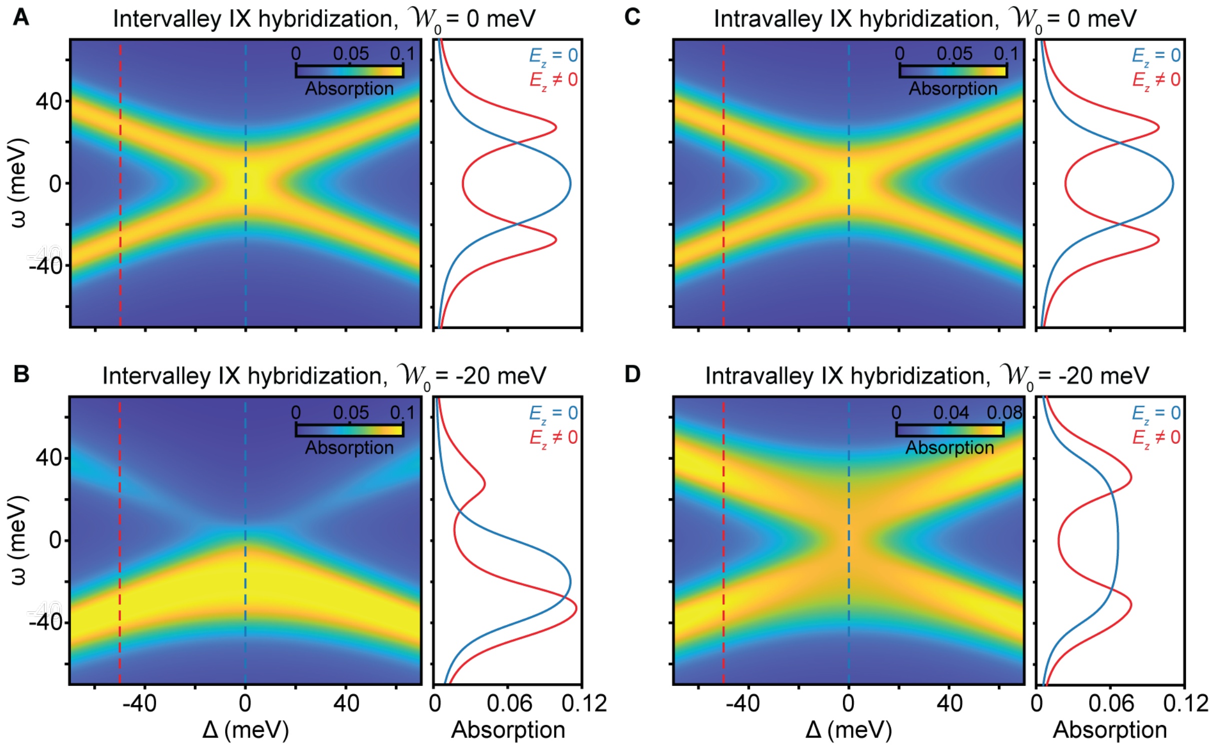

FIG. S11. Intravalley vs. intervalley exciton hybridization. **A,B** Assuming the case of intervalley hybridization, the simulated stochastic anti-crossing for $\mathcal{W}_0 = 0$ **A** and for $\mathcal{W}_0 = -20\,\text{meV}$ **B** illustrates that a nonzero $\mathcal{W}_0 \neq 0$ leads to an intensity asymmetry between the upper and lower exciton branches. Such asymmetry emerges due to an interference effect between the two intervalley excitons with the same AQNs. In contrast, for the case of intravalley hybridization **C,D**, the stochastic anti-crossing shows no such asymmetry even for $\mathcal{W}_0 \neq 0$ because intravalley interlayer excitons have opposite AQNs and, thus, cannot interfere. Here we fixed $\sigma = 20\,\text{meV}$.

IX. DATA PROCESSING

When one considers reflectivity properties of a TMD sample, it is natural to separate the background contribution $R_{\text{bg}}(\omega)$ from the excitonic resonances [8–10]:

$$R(\omega) \approx R_{\text{bg}}(\omega) - \text{Im}[e^{i\varphi(\omega)}(\chi_{\text{IX}}(\omega) + \chi_{\text{A}}(\omega))], \tag{S11}$$

where $\varphi(\omega)$ encodes the effects due to the interference between the background and excitonic parts. In analyzing the measured data, we employ Eqs. (S7) and (S8) to fit the interlayer exciton contribution and

$$\chi_{\text{A}}(\omega) = -\frac{d_{\text{A}}^2}{\omega - \omega_{\text{A}} + i\gamma_{\text{A}}} \tag{S12}$$

to fit the A-exciton contribution. While we are primarily interested in the IX-properties, we consider a rather large fitting energy range (about 100 meV) so that the A-exciton part cannot be fully disregarded – see Fig. S10. We let $R_{\text{bg}}(\omega) \approx R_0 + R_1(\omega - 2000\,\text{meV})$ to be linearly dependent on ω for $|\omega - 2000\,\text{meV}| \ll 2000\,\text{meV}$ (the choice of 2000 meV is close to the interlayer exciton energy at $E_z = 0$). In principle, $\varphi(\omega)$ can also depend on ω, but we verified that approximating $\varphi(\omega)$ as constant, denoted as φ_0, provides good fits to the data.

In our analysis, we fit the normalized reflectance data $\mathcal{S}(\omega) = (R_{\text{TMD}} - R_{\text{no-TMD}})/R_{\text{no-TMD}}$, using a similar fitting form as in Eq. (S11). Here, $R_{\text{no-TMD}}$ represents the measured reflectance from the graphite/hBN/hBN/graphite heterostructure, i.e., from the full stack but without the TMD part – it should reasonably well approximate R_{bg} in Eq. (S11).

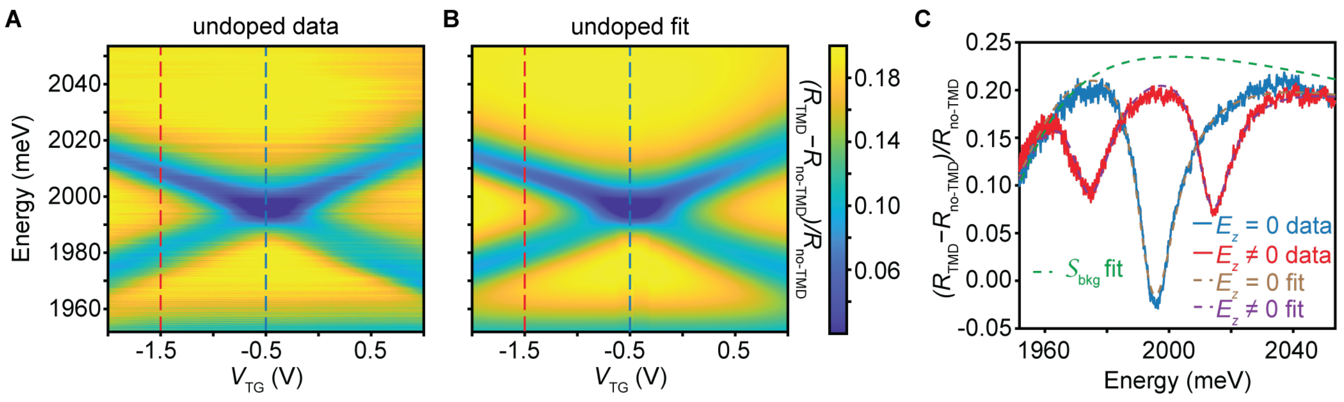

FIG. S12. Data processing when the sample is undoped. **A** and **B** show a back-to-back comparison of the measured (**A**) and fitted (**B**) electric-field sweeps, demonstrating excellent quantitative agreement. **C** This agreement is further corroborated by individual linecuts at $E_z = 0$ and $E_z \neq 0$. The green dashed line represents the fitted background, which includes the A-exciton contribution.

A. Fitting the intrinsic data

We use the intrinsic data to fix the three background parameters ($\mathcal{S}_0$, $\mathcal{S}_1$, and φ_0), which are assumed to be independent of applied voltages and, thus, electron doping. Here, each interlayer exciton is modeled by a Lorentzian as in Eq. (S12), with three parameters ($\omega_{T/B}$, $\gamma_{T/B}$, and $d_{T/B}$) that can vary with E_z (V_{TG}). The A-exciton parameters (d_A, ω_A, and γ_A) can depend on the electron density but not on the electric field E_z, acting as a correction to the background R_{bg}. Figure S12 demonstrates that this fitting approach accurately captures the intrinsic data, allowing us to confidently estimate the background parameters $\mathcal{S}_0$, $\mathcal{S}_1$, and φ_0.

B. Fitting the doped data

As discussed in Sec. VIII, we analyze interlayer excitons using full 2D reflectance maps and model IXs via Eqs. (S7) and (S8). To minimize the number of fitting parameters while capturing both the linear Stark effect and stochastic hybridization, we represent these excitons with six parameters: $\mathcal{W}_0$, σ, $d_T = d_B = d$, $\gamma_T = \gamma_B$, and both the bare interlayer exciton energy ω_0 and linear Stark shift, as encoded in d_z, are allowed to depend on the electron density. Figure S13 shows that this few-parameter fit reasonably well captures the measured signal. While not as perfect as in Fig. S12, this fit provides robust data processing (see below) by using significantly fewer parameters and still captures the essential physics. This fitting procedure is then used to analyze the experiment (see Fig. 2 of the main text).

Let us comment on the physical content of each of the six fitting parameters associated with the interlayer excitons:

- The mean coupling $\mathcal{W}_0$ encodes the intensity asymmetry between the upper and lower exciton branches.
- The stochastic variance σ describes the stochastic anti-crossing, which is most evident at $E_z = 0$.
- The rate γ corresponds to the excitonic linewidth for some appreciable $E_z \neq 0$.
- The transition dipole moment d encodes the exciton-photon coupling and IX oscillator strength.
- d_z is the effective IX out-of-plane dipole moment.
- ω_0 is the bare IX energy at $E_z = 0$.

C. Error bar analysis

In estimating error bars for the fitted mean coupling $\mathcal{W}_0$ and stochastic variance σ, we consider both experimental error and fitting error.

The experimental error arises primarily from two factors: i) the inherent variability in individual measured spectra, as each spectrum represents an average of several measurements, leading to a variance in the measured signal, and

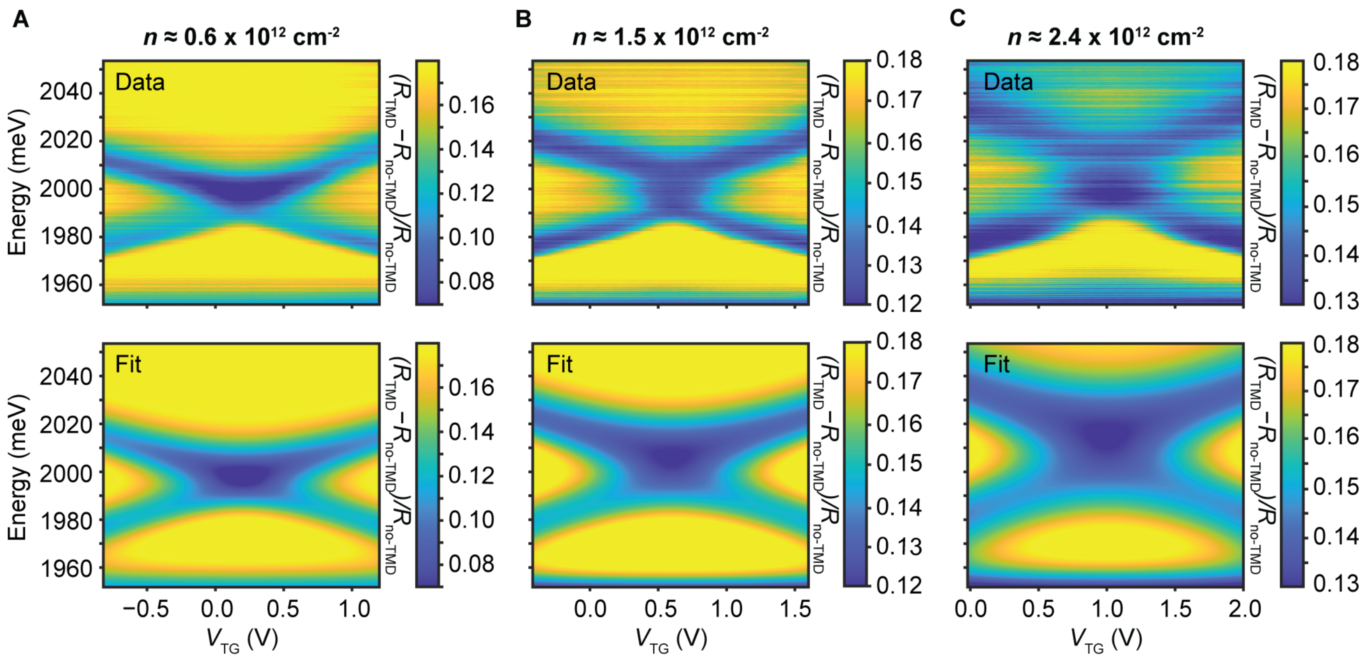

FIG. S13. Data processing when the sample is doped. **A**, **B**, **C** depict a back-to-back comparison of the measured (top panels) and fitted (bottom panels) electric-field sweeps at three representative dopings. This comparison demonstrates that the few-parameter fit is in reasonable quantitative agreement with the data, capturing both the linear Stark effect and the stochastic anti-crossing.

ii) the signal-to-noise ratio, which we assess by smoothing the spectrum using a Savitzky-Golay filter and extracting the variance relative to the smoothed spectrum. However, we find that the experimental error is negligible compared to the fitting error. A complication we face with our fits is the worsening signal-to-noise ratio as the temperature increases.

When using standard statistical tools, such as those based on confidence intervals (*lsqcurvefit* function in Matlab), we often obtain fitting errors that are unreasonably small. This situation arises because our few-parameter model is somewhat constrained, leading to an incidence of underfitting in the analysis of 2D reflectance maps [11, 12]. To estimate the fitting error bars for parameters like the stochastic variance σ, we then proceed as follows: we sweep σ around the optimal value σ^* while re-fitting the remaining parameters and evaluating the global least-squares error:

$$\mathcal{F}[\boldsymbol{\theta}] = \sum_{i,j} \left| \mathcal{S}(\omega_i, V_{\mathrm{TG},j}) - \mathcal{S}_{\mathrm{fit}}[\boldsymbol{\theta}](\omega_i, V_{\mathrm{TG},j}) \right|^2 , \tag{S13}$$

where $\boldsymbol{\theta}$ represents the vector of fitting parameters (in our case, these are three parameters associated with the A-exciton and six with the interlayer excitons). Figure S14A,B shows such scans for the stochastic variance σ (A) and the mean coupling $\mathcal{W}_0$ (B), where, as expected, the global error displays a minimum at the optimized values σ^* and $\mathcal{W}_0^*$, respectively. We define the error tolerance to be 5% above the global error minimum, leading to error bars that significantly better represent the measured data (Fig. 2 of the main text).

To provide more insight in how well the fitting model represents the measured signal, we perform similar but now 2D scans of the global error as in Fig. S14C,D. We find reasonably isotropic error contours indicative of i) the robustness of parameter estimates, ii) the consistency of our fitting, and iii) the independence of model parameters [11, 12]. These contours, thus, suggest that the model is well-suited to capturing the underlying data distribution and that the fitted parameters accurately represent the relationships between the model and the observed data.

X. THEORY FOR THE STOCHASTIC ANTI-CROSSING

In the main text, we argued that the experimental conditions (low temperatures, strong Coulomb interactions $r_s \simeq 10 - 20$, small interlayer separation $lk_F \ll 1$, and absence of electron tunneling) strongly suggest the presence of interlayer electron coherence [13–15]. Here, we further discuss how such coherence can lead to the robust emergence of the stochastic anti-crossing.

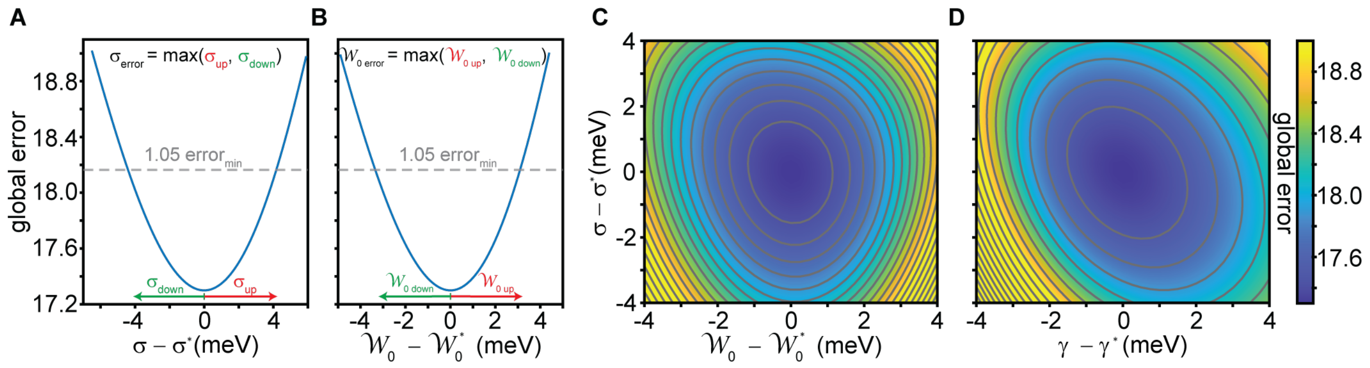

FIG. S14. Error bar analysis of the stochastic variance σ (**A**) and the mean coupling $\mathcal{W}_0$ (**B**). The error bars are estimated as follows: we scan σ (or $\mathcal{W}_0$) near its optimized value σ^* ($\mathcal{W}_0^*$), while the rest of the parameters are refitted, and plot the resulting global least-squares error function. To determine the error bar, we use an error tolerance of 5% above the global error minimum (indicated by dashed lines in **A** and **B**). **C** and **D** depict the global error function in the ($\mathcal{W}_0, \sigma$)-map (**C**) and (γ_0, σ)-map (**D**), respectively, illustrating that the global error is reasonably isotropic in the vicinity of the optimized parameters.

In TMDs, the valley degree of freedom allows for interlayer electron coherence with intravalley character, $\Delta_{\text{intra,K}}(\boldsymbol{k}) = \langle \hat{e}^\dagger_{\text{T,K}}(\boldsymbol{k})\hat{e}_{\text{B,K}}(\boldsymbol{k})\rangle \neq 0$, and/or intervalley character, $\Delta_{\text{inter}}(\boldsymbol{k}) = \langle \hat{e}^\dagger_{\text{T,K}}(\boldsymbol{k})\hat{e}_{\text{B,K}'}(\boldsymbol{k})\rangle \neq 0$ (see Fig. 4A of the main text). Here, $\hat{e}^\dagger_{\text{T,K}}(\boldsymbol{k})$ is the electron creation operator in the top K-valley, with $\boldsymbol{k}$ being the wave vector relative to the band bottom. For our discussion, we assume the system is spin-polarized but valley-depolarized, a situation expected in our finite magnetic-field measurements (see Fig. 3 of the main text). This assumption simplifies the analysis by omitting the electron spin, although including it is straightforward. The close energies of these two order parameters make it likely that both are present and interplay within the system, as further discussed below.

A. Intravalley interlayer exciton hybridization and crude estimates

Interlayer excitons serve as an optical probe of doped electrons; for this reason, we distinguish between the electron system with its microscopic interactions and the excitonic probe. Assuming the presence of interlayer electron coherence, we show here how this order parameter leads to interlayer exciton hybridization, consistent with our experimental findings, and discuss its stochastic behavior.

The absence of electron tunneling in MoS_2-homobilayers [16] indicates that the many-body electron Hamiltonian (approximately, see Sec. XI) commutes with the particle number operators $\hat{N}_{\text{T}}$ and $\hat{N}_{\text{B}}$ in each layer. Interlayer electron coherence corresponds to the spontaneous breaking of layer U(1) symmetry (in Sec. XI, we discuss that this U(1) symmetry is weakly broken down to $\mathbb{Z}_2$), where the symmetry-broken state is not an eigenstate of $\hat{N}_{\text{T}} - \hat{N}_{\text{B}}$. This order parameter can be thought of as pseudospin ferromagnetism with the layer pseudospin pointing in-plane (see, for instance, Ref. [13]).

The key idea of the hybridization mechanism we propose in Fig. 4B of the main text is that the presence of the intravalley coherence mediates strong electron tunneling-like processes. To illustrate this, we write the density-density interlayer Coulomb interaction between conduction-band electrons as (scattered electrons remain in the same valley and layer):

$$\hat{H}_l = \frac{1}{\mathcal{A}} \sum_{\boldsymbol{k},\boldsymbol{k}',\boldsymbol{q}} \sum_{\alpha,\beta=\text{K,K}'} V_l(q)\hat{e}^\dagger_{\text{T},\alpha}(\boldsymbol{k}+\boldsymbol{q})\hat{e}_{\text{T},\alpha}(\boldsymbol{k})\hat{e}^\dagger_{\text{B},\beta}(\boldsymbol{k}')\hat{e}_{\text{B},\beta}(\boldsymbol{k}'+\boldsymbol{q}), \tag{S14}$$

where $V_l(q)$ is the interlayer Coulomb potential and $\mathcal{A}$ is the area of the sample. For estimates, we use $V_l(q) = 2\pi e^2 e^{-ql}/(\varepsilon q)$ with $\varepsilon \approx 3.76$ – this form might slightly overestimate the strength of interlayer interactions as the TMD permittivity is larger than that of hBN [16]. The presence of a nonzero order parameter, $\Delta_{\text{intra,K}}(\boldsymbol{k}) = \langle \hat{e}^\dagger_{\text{T,K}}(\boldsymbol{k})\hat{e}_{\text{B,K}}(\boldsymbol{k})\rangle \neq 0$, results in an effective electron tunneling-like term (we write only the processes in the K-valley):

$$-\sum_{\boldsymbol{k}} t_{\boldsymbol{k}}\hat{e}^\dagger_{\text{B,K}}(\boldsymbol{k})\hat{e}_{\text{T,K}}(\boldsymbol{k}) + \text{h.c.}, \tag{S15}$$

where the effective tunneling rate is set by the order parameter:

$$t_{\boldsymbol{k}} = \frac{1}{\mathcal{A}} \sum_{\boldsymbol{q}} V_l(\boldsymbol{q}) \Delta_{\text{intra,K}}(\boldsymbol{k}+\boldsymbol{q}). \tag{S16}$$

Let us note that a small but finite electron tunneling, which explicitly breaks the U(1) layer symmetry, would imprint the phase on the order parameter, much like a small magnetic field in a ferromagnet polarizes spins along its direction. In spin systems with strong ferromagnetic correlations, a small magnetic field induces significant spin polarization. Analogously, in our case, even weak interlayer electron tunneling is expected to enhance tunneling conductance. This effect, a definitive signature of interlayer electron coherence, has been experimentally established in conventional quantum Hall bilayers [15, 17–21].

The layer separation in our system is only a few angstroms, making the effective electron tunneling-like processes in Eq. (S16) strong. Assuming perfect Hartree-Fock correlations with $\Delta_{\text{intra,K}}(k) \simeq n_F(k)$ (n_F is the Fermi-Dirac distribution function), we estimate $t_{\boldsymbol{k}=\boldsymbol{0}} \simeq e^2\sqrt{\pi n}/\varepsilon \simeq 96\,\text{meV}$ and $t_{k=1/a_X} \simeq 36\,\text{meV}$ for four electron bands (corresponding to the spin-polarized case as in Fig. 3 of the main text), $n = 2 \times 10^{12}\,\text{cm}^{-2}$, $a_X = 3\,\text{nm}$, and $T = 0\,\text{K}$ – see also Sec. X C, where we detail our self-consistent Hartree-Fock analysis.

The significance of such tunneling-like electron processes is that they give rise to the hybridization of, for example, IX_T- and A_B-excitons – see Fig. 4B (left) of the main text. This coupling could be estimated as (we note that $l \ll a_X$):

$$t_{\text{IX}_\text{T}\leftrightarrow \text{A}_\text{B}} \simeq \int \frac{d^2\boldsymbol{k}}{(2\pi)^2} t_{\boldsymbol{k}} \Psi_A^*(\boldsymbol{k}) \Psi_X(\boldsymbol{k}) \simeq 85\,\text{meV for } n = 2\times 10^{12}\,\text{cm}^{-2}, \tag{S17}$$

where we substituted for the exciton wave-functions $\Psi_A(\boldsymbol{q}) \approx \Psi_X(\boldsymbol{q}) \approx 2\sqrt{2\pi} a_X/((qa_X)^2+1)^{3/2}$. Given our assumptions, the value in Eq. (S17) is likely an overestimate, but it nevertheless underscores the importance of the processes we propose. At the same time, the A_B-exciton couples to the IX_B-state via the two-step process shown in Fig. 4B (middle and right panels) of the main text, with experimental evidence supporting this effect [7] and a coupling strength of about ~ 4 meV. All three processes in Fig. 4B of the main text combined lead to the intravalley interlayer exciton hybridization (we again write only the K-valley terms):

$$\hat{H}_{\text{intravalley}} = \delta\mathcal{W}[\Delta_{\text{intra,K}}] \hat{X}^\dagger_{\text{B,K}} \hat{X}_{\text{T,K}} + \text{h.c.} \tag{S18}$$

Using second-order perturbation theory, the coupling $\delta\mathcal{W}[\Delta_{\text{intra,K}}]$ between the interlayer excitons can then be estimated as $85\,\text{meV} \times 4\,\text{meV}/70\,\text{meV} \simeq 5\,\text{meV}$ for $n = 2\times 10^{12}\,\text{cm}^{-2}$ (here, 70 meV is the energy difference between IX- and A-excitons). While this estimate is rather crude – as we (i) assumed perfect Hartree-Fock correlations, (ii) considered only the lowest energy intermediate exciton states (we note that the $2s$ A-exciton, though having a small oscillator strength, is energetically closer to the interlayer excitons, see also Sec. XI), and (iii) used perturbation theory to relate the electronic order parameter to interlayer exciton hybridization – we find that the estimated value is comparable to the measured ones (Fig. 2E of the main text), suggesting that the proposed hybridization mechanism is realistic.

B. Additional symmetry considerations for MoS_2-homobilayers

In contrast to conventional semiconductors with a single electron valley (assuming the electron system is spin-polarized), MoS_2-homobilayers feature not just a single but two distinct K- and K'-valleys (when the electron spin can be disregarded, these valleys are approximately degenerate), which bring in an additional spin-1/2-like degree of freedom. The effective microscopic Hamiltonian, consisting of the electron kinetic energy (with approximately parabolic dispersion) in each of the valleys and Coulomb interactions, now commutes with the particle number operators $\hat{N}^\alpha_{\text{T/B}} = \mathcal{A}^{-1}\sum_{\boldsymbol{k}} \hat{e}^\dagger_{\text{T/B},\alpha}(\boldsymbol{k})\hat{e}_{\text{T/B},\alpha}(\boldsymbol{k})$ in each layer and each valley $\alpha \in \{\text{K},\text{K}'\}$. The valley degree of freedom suggests the introduction of the order parameter as:

$$\hat{e}^\dagger_{\text{T},\alpha}(\boldsymbol{k})\hat{e}_{\text{B},\beta}(\boldsymbol{k}) \to \hat{\Delta}_0(\boldsymbol{k})\tau^0_{\alpha\beta} + [\hat{\Delta}_x(\boldsymbol{k})\tau^x_{\alpha\beta} + \hat{\Delta}_y(\boldsymbol{k})\tau^z_{\alpha\beta} + \hat{\Delta}_z(\boldsymbol{k})\tau^z_{\alpha\beta}], \tag{S19}$$

where $\hat{\Delta}_a(\boldsymbol{k}) \equiv \frac{1}{2}\hat{e}^\dagger_{\text{T},\alpha}(\boldsymbol{k})\tau^a_{\alpha\beta}\hat{e}_{\text{B},\beta}(\boldsymbol{k})$, $a \in \{0,x,y,z\}$, and τ^a are the Pauli matrices in the valley-space. The K-valley electron coherence, which determines the K-valley interlayer exciton hybridization in Eq. (S18), see Sec. X A, is then written as $\Delta_{\text{intra,K}}(\boldsymbol{k}) = \langle\hat{\Delta}_0(\boldsymbol{k})\rangle + \langle\hat{\Delta}_z(\boldsymbol{k})\rangle$. We note that the component $\hat{\Delta}_0$ transforms trivially (as a scalar) under the global SU(2) valley rotations, while the vector components $(\hat{\Delta}_x, \hat{\Delta}_y, \hat{\Delta}_z)$ transform as an SU(2) triplet. Therefore, if the electronic ground state develops $\langle\hat{\Delta}_0\rangle \neq 0$ only, it breaks the original U(1)×SU(2) symmetry – here, the U(1)

part is associated with the operator $\hat{N}_{\rm T}^{\rm tot} - \hat{N}_{\rm B}^{\rm tot} = \hat{N}_{\rm T}^{\rm K} + \hat{N}_{\rm T}^{\rm K'} - \hat{N}_{\rm B}^{\rm K} - \hat{N}_{\rm B}^{\rm K'}$, while the SU(2) part is associated with valley pseudospin rotations – down to SU(2). On the other hand, the development of a nonzero vector component breaks both the U(1) and SU(2) parts of this U(1)×SU(2) symmetry.

This effective microscopic description, where the valley index is analogous to a spin index, implies that the system can be thought of as effectively translationally invariant (noting that in the sample, intervalley correlations actually carry the momentum $\boldsymbol{K} - \boldsymbol{K}'$). For this reason, in Eq. (S19), we consider both electron operators to have the same momentum. In other words, we assume that the ground state can give a nonzero expectation value $\langle \hat{e}^\dagger_{{\rm T},\alpha}(\boldsymbol{k})\hat{e}_{{\rm B},\beta}(\boldsymbol{k}')\rangle \neq 0$ only if $\boldsymbol{k} = \boldsymbol{k}'$. With this in mind, we write the reduced [22] (corresponding to $\boldsymbol{k} = \boldsymbol{k}'$ in Eq. (S14)) interlayer Coulomb interaction (S14) as:

$$\hat{H}_l \to -\frac{2}{\mathcal{A}}\sum_{\boldsymbol{k},\boldsymbol{k}'} V_l(|\boldsymbol{k}-\boldsymbol{k}'|)[\hat{\Delta}_0^\dagger(\boldsymbol{k})\hat{\Delta}_0(\boldsymbol{k}') + \hat{\Delta}_x^\dagger(\boldsymbol{k})\hat{\Delta}_x(\boldsymbol{k}') + \hat{\Delta}_y^\dagger(\boldsymbol{k})\hat{\Delta}_y(\boldsymbol{k}') + \hat{\Delta}_z^\dagger(\boldsymbol{k})\hat{\Delta}_z(\boldsymbol{k}')], \tag{S20}$$

and this form is clearly U(1)×SU(2) symmetric. This form also indicates that the intravalley and intervalley electron coherences stand on equal footing in our system.

At the same time, from the perspective of intravalley interlayer excitons, the coupling in Eq. (S18) requires the explicit presence of a nonzero order parameter. Indeed, within the perturbation theory discussed above, we get

$$\delta\hat{\mathcal{W}}_{\rm intra,K} \propto t_h \int \frac{d^2\boldsymbol{k}}{(2\pi)^2}\int \frac{d^2\boldsymbol{q}}{(2\pi)^2}\Psi_A^*(\boldsymbol{k})\Psi_X(\boldsymbol{k})V_l(\boldsymbol{q})[\hat{\Delta}_0(\boldsymbol{k}+\boldsymbol{q}) + \hat{\Delta}_z(\boldsymbol{k}+\boldsymbol{q})], \tag{S21}$$

where t_h is the hole tunneling rate, on the order of a few tens of meV in MoS_2-homobilayers. (From the symmetry perspective, holes are allowed to tunnel as the valence bands have the same AQNs, see also the right panel of Fig. 4B in the main text.) The expression (S21) indicates that $\delta\hat{\mathcal{W}}_{\rm intra,K}$ is sensitive to both the order parameter amplitude and phase, thereby inheriting spatial inhomogeneities due to statistical fluctuations of the order parameter phase. Consequently, the model in Eq. (S18) should be extended to account for spatial dependence. In the experiment, this coupling is spatially averaged over the optical spot size, which we expect to be much larger than the phase coherence length (see also Sec. V). As a result, the spatial average $\langle\delta\mathcal{W}[\Delta_{\rm intra,K}]\rangle \approx 0$ vanishes, but the variance σ can be significant, manifesting as the stochastic anti-crossing. This explains the experimental observations, as elaborated in the main text.

We comment that MoS_2-homobilayers possess $\mathcal{C}_3$-rotationally rotational symmetry, assigning opposite AQNs to interlayer excitons within the same valley. As a result, there is no Hamiltonian term that directly couples such two excitons (disorder could potentially play a role, but as discussed in the main text, it is not expected to be dominant). These excitons can hybridize when the system breaks this symmetry, specifically through intravalley interlayer electron coherence. In other words, the coupling in Eq. (S18) serves as a probe of this order parameter. We also note that because these two excitons have opposite AQNs, their hybridization cannot explain the small intensity asymmetry observed between the lower and upper exciton branches – see Fig. S10 and Sec. VIII. We address this question further in the following section.

C. Self-consistent Hartree-Fock analysis

We conclude this section by presenting our self-consistent Hartree-Fock analysis, which reasonably captures the observations in Fig. 2E,F of the main text. These calculations extend the single-band analysis in Refs. [13, 14] to the case of two K- and K'-valleys relevant for TMDs. Specifically, we consider the following microscopic Hamiltonian (neglecting electron spin and, as such, the small spin-orbit coupling):

$$\hat{H} = \sum_{\boldsymbol{k}}\sum_{l,v}\frac{k^2}{2m^*}\hat{e}^\dagger_{lv}(\boldsymbol{k})\hat{e}_{lv}(\boldsymbol{k}) + \frac{1}{2\mathcal{A}}\sum_{\boldsymbol{k},\boldsymbol{k}',\boldsymbol{q}}\sum_{l}\sum_{vv'}V(q)\hat{e}^\dagger_{lv}(\boldsymbol{k}+\boldsymbol{q})\hat{e}^\dagger_{lv'}(\boldsymbol{k}'-\boldsymbol{q})\hat{e}_{lv'}(\boldsymbol{k}')\hat{e}_{lv}(\boldsymbol{k})$$
$$+\frac{1}{\mathcal{A}}\sum_{\boldsymbol{k},\boldsymbol{k}',\boldsymbol{q}}\sum_{vv'}V_l(q)\hat{e}^\dagger_{{\rm T}v}(\boldsymbol{k}+\boldsymbol{q})\hat{e}^\dagger_{{\rm B}v'}(\boldsymbol{k}'-\boldsymbol{q})\hat{e}_{{\rm B}v'}(\boldsymbol{k}')\hat{e}_{{\rm T}v}(\boldsymbol{k}), \tag{S22}$$

where $l \in \{{\rm T},{\rm B}\}$ is the layer index, $v \in \{K, K'\}$ is the valley index, and m^* is the effective electron mass.

Within the Hartree-Fock approximation, and in the strongly-interacting regime relevant to the experiment ($1 \ll r_s$), the ground-state wave function with $\langle\hat{\Delta}_0\rangle \neq 0$ is given by (analogous to the $|S_2\rangle$-state in the single-band case [13, 14]):

$$|\psi_0\rangle = \prod_{k\leq k_F}\frac{1}{2}(\hat{e}^\dagger_{\rm T,K}(\boldsymbol{k}) + \hat{e}^\dagger_{\rm B,K}(\boldsymbol{k}))(\hat{e}^\dagger_{\rm T,K'}(\boldsymbol{k}) + \hat{e}^\dagger_{\rm B,K'}(\boldsymbol{k}))\,|0\rangle\,, \tag{S23}$$

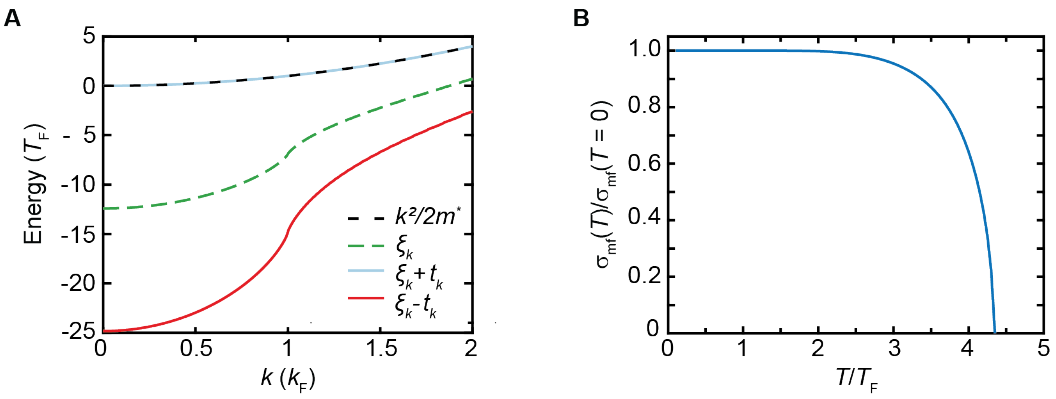

FIG. S15. Self-consistent Hartree-Fock simulation at $n \approx 1.3 \times 10^{12}\,\mathrm{cm}^{-2}$. **A** Modification of electron dispersion by the order parameter. **B** Temperature dependence.

where $k_F = \sqrt{2\pi n}$. For future reference, we define $T_F = k_F^2/(2m^*) = \pi n/m^* = (2/r_s^2)\mathrm{Ry}^*$, where $r_s = 1/(a^*\sqrt{\pi n})$, $\mathrm{Ry}^* \equiv e^2/(2a^*\varepsilon) = 1/(2m^*(a^*)^2)$ is the Rydberg energy, and $a^* \equiv \varepsilon/(m^*e^2)$ is the Bohr radius (estimated to be $a^* \simeq 0.3\,\mathrm{nm}$ and $\mathrm{Ry}^* \simeq 530\,\mathrm{meV}$).

We propose that this many-body ground state wave funcion (S23) can qualitatively explain our low-temperature measurements shown in Fig. 2E of the main text. To support this, we evaluate the expression in Eq. (S17), which captures the effective hybridization strength between the $\mathrm{IX_T}$- and A_B-excitons:

$$t_{\mathrm{IX_T}\leftrightarrow \mathrm{A_B}} \simeq \int \frac{d^2\boldsymbol{k}}{(2\pi)^2}\frac{8\pi a_X^2}{(1+(ka_X)^2)^3}\int \frac{d^2\boldsymbol{q}}{(2\pi)^2}V_l(\boldsymbol{q})\langle \hat{e}^\dagger_{\mathrm{T,K}}(\boldsymbol{k}+\boldsymbol{q})\hat{e}_{\mathrm{B,K}}(\boldsymbol{k}+\boldsymbol{q})\rangle \tag{S24}$$

$$\simeq \int \frac{d^2\boldsymbol{k}}{(2\pi)^2}\frac{8\pi a_X^2}{(1+(ka_X)^2)^3}\frac{2\pi e^2}{k\varepsilon}\int \frac{d^2\boldsymbol{q}}{(2\pi)^2}\langle \hat{e}^\dagger_{\mathrm{T,K}}(\boldsymbol{q})\hat{e}_{\mathrm{B,K}}(\boldsymbol{q})\rangle \tag{S25}$$

$$= \frac{3\pi^2 e^2 a_X}{2\varepsilon}\int \frac{d^2\boldsymbol{q}}{(2\pi)^2}\langle \hat{e}^\dagger_{\mathrm{T,K}}(\boldsymbol{q})\hat{e}_{\mathrm{B,K}}(\boldsymbol{q})\rangle = \frac{3\pi^2 e^2 a_X}{8\varepsilon}n, \tag{S26}$$

where in the second identity, we have used $k_F \ll a_X^{-1} \ll l^{-1}$, a condition that further implies that the rate $t_{\mathrm{IX_T}\leftrightarrow \mathrm{A_B}} \propto \Delta_0(\boldsymbol{r}=0)$ is approximately set by the local value of the order parameter $\Delta_0(\boldsymbol{r})$. Our mean-field analysis neglects spatial inhomogeneities in the order parameter due to phase fluctuations. Thus, using Eq. (S26) and perturbation theory for interlayer exciton hybridization described above, our Hartree-Fock analysis estimates the stochastic variance:

$$\sigma_{\mathrm{mf}} = C\int \frac{d^2\boldsymbol{q}}{(2\pi)^2}\langle \hat{e}^\dagger_{\mathrm{T,K}}(\boldsymbol{q})\hat{e}_{\mathrm{B,K}}(\boldsymbol{q})\rangle. \tag{S27}$$

where in our approach, C is a phenomenological parameter that may depend on temperature T. Notably, at low temperatures, σ_{mf} is approximately proportional to the electron density n, Eq. (S26), consistent with our measurements in Fig. 2E of the main text (dashed line), which were used to determine C. Let us comment that while the Hartree-Fock approximation might underestimate the role of low-momenta fluctuations, it should reasonably well capture local properties, particularly the stochastic variance σ.

To explore finite-temperature effects within the same mean-field approximation, where the many-body density matrix is assumed to be Gaussian, we introduce the covariance matrix:

$$\Gamma^{ll'}_{vv'}(\boldsymbol{k}) \equiv \langle \hat{e}^\dagger_{lv}(\boldsymbol{k})\hat{e}_{l'v'}(\boldsymbol{k})\rangle. \tag{S28}$$

For the state in Eq. (S23), the covariance matrix reads:

$$\Gamma_{\boldsymbol{k}}\Big|_{k\le k_F} = \frac{1}{2}\begin{bmatrix}\mathbb{1}_{2\times2} & \mathbb{1}_{2\times2}\\ \mathbb{1}_{2\times2} & \mathbb{1}_{2\times2}\end{bmatrix}. \tag{S29}$$

We note that this matrix satisfies the purity condition $\Gamma_{\boldsymbol{k}}^2 = \Gamma_{\boldsymbol{k}}$, consistent with the fact that the density matrix at $T=0$ is pure. The energy expectation value is then understood as:

$$E[\Gamma] = \sum_{\boldsymbol{k}}\frac{k^2}{2m^*}\mathrm{tr}(\Gamma(\boldsymbol{k})) - \frac{1}{2\mathcal{A}}\sum_{\boldsymbol{k},\boldsymbol{k}'}\sum_{l}\sum_{vv'}V(\boldsymbol{k}-\boldsymbol{k}')\Gamma^{ll}_{vv'}(\boldsymbol{k})\Gamma^{ll}_{v'v}(\boldsymbol{k}') - \frac{1}{\mathcal{A}}\sum_{\boldsymbol{k},\boldsymbol{k}'}\sum_{vv'}V_l(\boldsymbol{k}-\boldsymbol{k}')\Gamma^{\mathrm{TB}}_{vv'}(\boldsymbol{k})\Gamma^{\mathrm{BT}}_{v'v}(\boldsymbol{k}'), \tag{S30}$$

where the first term represents the kinetic energy, and the last two terms correspond to the exchange energies associated with intra- and interlayer electron interactions, respectively. The electrostatic energy is not included as it vanishes for the balanced symmetric case of equal densities considered here [13, 14, 23]. The presence of a finite out-of-plane electric field $E_z \neq 0$ would result in a density imbalance between the two layers, which generically suppresses the order parameter (see, for instance, Ref. [14]). Here, for the symmetric case $E_z = 0$, we further assume that (i) all four conduction bands (two valleys and two layers) are equally populated (consistent with the magnetic field measurements discussed in the main text) and (ii) there is no intralayer intervalley coherence. Under these assumptions, and using similar notation as above, the covariance matrix takes the form:

$$\Gamma(\boldsymbol{k}) = n_{\boldsymbol{k}}\mathbb{1}_{4\times4} + \begin{bmatrix} \mathbb{0}_{2\times2} & \Delta_0(\boldsymbol{k})\tau^0 + \Delta_x(\boldsymbol{k})\tau^x + \Delta_y(\boldsymbol{k})\tau^y + \Delta_z(\boldsymbol{k})\tau^z \\ \Delta_0^*(\boldsymbol{k})\tau^0 + \Delta_x^*(\boldsymbol{k})\tau^x + \Delta_y^*(\boldsymbol{k})\tau^y + \Delta_z^*(\boldsymbol{k})\tau^z & \mathbb{0}_{2\times2} \end{bmatrix}, \quad \text{(S31)}$$

where $\Delta_a(\boldsymbol{k}) = \frac{1}{2}\mathrm{tr}(\Gamma^{\mathrm{TB}}(\boldsymbol{k})\tau^a)$. For such states, the energy expectation value is further given by, cf. Eq. (S20):

$$E[\Gamma] = \sum_{\boldsymbol{k}} \frac{2k^2 n_{\boldsymbol{k}}}{m^*} - \frac{2}{\mathcal{A}}\sum_{\boldsymbol{k},\boldsymbol{k}'} V(\boldsymbol{k}-\boldsymbol{k}')n_{\boldsymbol{k}}n_{\boldsymbol{k}'}$$
$$- \frac{2}{\mathcal{A}}\sum_{\boldsymbol{k},\boldsymbol{k}'} V_l(\boldsymbol{k}-\boldsymbol{k}')[\Delta_0^*(\boldsymbol{k}')\Delta_0(\boldsymbol{k}) + \Delta_x^*(\boldsymbol{k}')\Delta_x(\boldsymbol{k}) + \Delta_y^*(\boldsymbol{k}')\Delta_y(\boldsymbol{k}) + \Delta_z^*(\boldsymbol{k}')\Delta_z(\boldsymbol{k})]. \quad \text{(S32)}$$

This expression reflects that the Hamiltonian in Eq. (S22) is U(1)×SU(2) symmetric, as discussed above.

The mean-field Hamiltonian $\hat{H}_{\mathrm{MF}} = \sum_{\boldsymbol{k}} \hat{\Psi}_{\boldsymbol{k}}^\dagger h(\boldsymbol{k})\hat{\Psi}_{\boldsymbol{k}}$, where $\hat{\Psi}_{\boldsymbol{k}} \equiv (\hat{e}_{\mathrm{T,K}}(\boldsymbol{k}), \hat{e}_{\mathrm{T,K'}}(\boldsymbol{k}), \hat{e}_{\mathrm{B,K}}(\boldsymbol{k}), \hat{e}_{\mathrm{B,K'}}(\boldsymbol{k}))^{\mathrm{T}}$, is derived from Eq. (S30) by evaluating the variational derivative $h_{vv'}^{ll'}(\boldsymbol{k}) = \delta E[\Gamma]/\delta\Gamma_{vv'}^{ll'}(\boldsymbol{k})$ and can be expressed as:

$$h_{vv'}^{ll'}(\boldsymbol{k}) = \frac{k^2}{2m^*}\delta_{ll'}\delta_{vv'} - \frac{\delta_{ll'}}{\mathcal{A}}\sum_{\boldsymbol{k}'} V(\boldsymbol{k}-\boldsymbol{k}')\Gamma_{v'v}^{ll}(\boldsymbol{k}') - \frac{(1-\delta_{ll'})}{\mathcal{A}}\sum_{\boldsymbol{k}'} V_l(\boldsymbol{k}-\boldsymbol{k}')\Gamma_{v'v}^{l'l}(\boldsymbol{k}'). \quad \text{(S33)}$$

Without loss of generality, and as follows from Eq. (S32), we can consider states that can have $\Delta_0(\boldsymbol{k}) \neq 0$ only (we also fix $\Delta_0(\boldsymbol{k})$ to be real so that the pseudospin points in-plane, along the x-axis), in which case the mean-field Hamiltonian is written as:

$$h(\boldsymbol{k}) = \xi_{\boldsymbol{k}}\mathbb{1}_{4\times4} - t_{\boldsymbol{k}}\begin{bmatrix} \mathbb{0}_{2\times2} & \mathbb{1}_{2\times2} \\ \mathbb{1}_{2\times2} & \mathbb{0}_{2\times2} \end{bmatrix}, \quad \text{(S34)}$$

where

$$\xi_{\boldsymbol{k}} = \frac{k^2}{2m^*} - \frac{1}{\mathcal{A}}\sum_{\boldsymbol{k}'} V(\boldsymbol{k}-\boldsymbol{k}')n_{\boldsymbol{k}'}, \qquad t_{\boldsymbol{k}} = \frac{1}{\mathcal{A}}\sum_{\boldsymbol{k}'} V(\boldsymbol{k}-\boldsymbol{k}')\Delta_0(\boldsymbol{k}'). \quad \text{(S35)}$$

We write the eigenvectors of Eq. (S34) as:

$$\begin{bmatrix} \hat{\psi}_{1,-}(\boldsymbol{k}) \\ \hat{\psi}_{2,-}(\boldsymbol{k}) \\ \hat{\psi}_{1,+}(\boldsymbol{k}) \\ \hat{\psi}_{2,+}(\boldsymbol{k}) \end{bmatrix} = \frac{1}{\sqrt{2}}\begin{bmatrix} 1 & 0 & 1 & 0 \\ 0 & 1 & 0 & 1 \\ 1 & 0 & -1 & 0 \\ 0 & 1 & 0 & -1 \end{bmatrix}\begin{bmatrix} \hat{e}_{\mathrm{T,K}}(\boldsymbol{k}) \\ \hat{e}_{\mathrm{T,K'}}(\boldsymbol{k}) \\ \hat{e}_{\mathrm{B,K}}(\boldsymbol{k}) \\ \hat{e}_{\mathrm{B,K'}}(\boldsymbol{k}) \end{bmatrix}, \quad \text{(S36)}$$

and the corresponding eigenvalues are $\xi_{\boldsymbol{k}} - t_{\boldsymbol{k}}$, $\xi_{\boldsymbol{k}} - t_{\boldsymbol{k}}$, $\xi_{\boldsymbol{k}} + t_{\boldsymbol{k}}$, and $\xi_{\boldsymbol{k}} + t_{\boldsymbol{k}}$, respectively – see Fig. S15A. Self-consistency then implies:

$$n_{\boldsymbol{k}} = \frac{1}{2}[n_F(\xi_{\boldsymbol{k}} - t_{\boldsymbol{k}}) + n_F(\xi_{\boldsymbol{k}} + t_{\boldsymbol{k}})], \qquad \Delta_0(\boldsymbol{k}) = \frac{1}{2}[n_F(\xi_{\boldsymbol{k}} - t_{\boldsymbol{k}}) - n_F(\xi_{\boldsymbol{k}} + t_{\boldsymbol{k}})], \quad \text{(S37)}$$

where $n_F(\varepsilon) = [1 + \exp((\varepsilon - \mu)/T)]^{-1}$ is the Fermi-Dirac distribution function, and the chemical potential μ is set by the total density $n = 2\int \frac{d^2\boldsymbol{k}}{(2\pi)^2}[n_F(\xi_{\boldsymbol{k}} - t_{\boldsymbol{k}}) + n_F(\xi_{\boldsymbol{k}} + t_{\boldsymbol{k}}))]$.

We solve Eqs. (S35) and (S37) numerically, with the results shown in Fig. S15B using experimental parameters at $n = 1.3 \times 10^{12}\,\mathrm{cm}^{-2}$, i.e, as in Fig. 2F of the main text. The Hartree-Fock approximation predicts a critical temperature of $T_c \simeq 4.35T_F \simeq 225\,\mathrm{K}$; however, this value should be considered an upper bound, as this method tends to underestimate the role of low-momenta fluctuations. By rescaling the temperature to match the experimental critical temperature $T_c = 75\,\mathrm{K}$ and using the experimentally determined value of C (assumed to be temperature independent), we achieve reasonable agreement between the theory and the data, as shown in Fig. 2F of the main text. This analysis suggests that the disappearance of the stochastic anti-crossing can be understood as the order parameter amplitude is suppressed with increasing T until it eventually melts.

XI. INTERVALLEY INTERLAYER EXCITON HYBRIDIZATION, WEAK SYMMETRY BREAKING, AND FERMI SEA FLUCTUATIONS

Our theory in Sec. X provides an interpretation of essentially all the experimental features except the small asymmetry between the lower and upper exciton branches – see, for instance, Fig. S10. In Sec. VIII, we argued that this asymmetry is indicative of intervalley interlayer exciton hybridization (as opposed to intravalley exciton hybridization discussed in Sec. X), which have the same AQNs. In the experiment, this asymmetry is associated with the development of small mean value $\mathcal{W}_0 \neq 0$, which has large error bars – see Fig. 2E,F of the main text. Furthermore, in Sec. V we further experimentally argue that this mean value $\mathcal{W}_0 \neq 0$ does not originate from order parameter phase coherence.

Because these excitons – such as $X_{\mathrm{B,K}}$ and $X_{\mathrm{T,K'}}$ (depicted in red in Fig. 1B of the main text) – have the same AQNs, there is no symmetry argument preventing their direct hybridization even in the absence of doped electrons. However, such a direct coupling is expected to be weak because the involved processes require both exciton electron and hole layer and valley switching. This expectation aligns with the experiment in Fig. 2E, which indicates that $|\mathcal{W}_0| \lesssim 2\,\mathrm{meV}$ for $n = 0$. We note that the interlayer excitons have rather large linewidths on the order of 10 meV, making it difficult to resolve a small possible hybridization. It is plausible that doped carriers could amplify this coupling through simple processes that do not involve the exotic physics discussed in the preceding section. To illustrate this expectation, in this section, we provide one such mechanism based on dynamical Fermi liquid fluctuations (we also mentioned in the main text that polaronic dressing might be important for the intervalley scenario as well). Let us remark that while the existence of such dynamical processes can explain the weak exciton intensity asymmetry, they cannot account for the static stochastic variance σ.

To understand how intervalley interlayer excitons could hybridize, we introduce processes termed 'hole flip' and 'electron flip', both corresponding to layer switching and scattering across the TMD Brillouin zone – see Fig. S16. The hole flip, shown in Fig. S16 (left), can occur through the simultaneous scattering of the hole of the $X_{\mathrm{T,K'}}$-exciton from the bottom K'-valley to the top K-valley and a Fermi sea electron. In such scattering processes, the total momentum is conserved, and the spin and AQN of the Fermi sea electron remain unchanged, resulting in the two possibilities depicted in Fig. S16 (left). Figure S16 (right) shows that similar Fermi sea scatterings can give rise to the electron flip, allowing us to write the following bare microscopic Hamiltonian:

$$\hat{H}_{\mathrm{int}} = \frac{V_a}{\mathcal{A}} \sum_{\boldsymbol{k},\boldsymbol{k}',\boldsymbol{q}} (\hat{\mathcal{F}}^{(e)}_{\boldsymbol{k}+\boldsymbol{q},\boldsymbol{k}} \hat{e}^\dagger_{\mathrm{B,K},\uparrow}(\boldsymbol{k}'-\boldsymbol{q}) \hat{e}_{\mathrm{T,K'},\uparrow}(\boldsymbol{k}') - \hat{\mathcal{F}}^{(h)}_{\boldsymbol{k}+\boldsymbol{q},\boldsymbol{k}} \hat{h}^\dagger_{\mathrm{T,K},\downarrow}(\boldsymbol{k}'-\boldsymbol{q}) \hat{h}_{\mathrm{B,K'},\downarrow}(\boldsymbol{k}')) + \mathrm{h.c.}, \tag{S38}$$

$$\hat{\mathcal{F}}^{(e)}_{\boldsymbol{k}_1,\boldsymbol{k}_2} \equiv \sum_\sigma [\hat{e}^\dagger_{\mathrm{B,K'},\sigma}(\boldsymbol{k}_1) \hat{e}_{\mathrm{T,K},\sigma}(\boldsymbol{k}_2) + \hat{e}^\dagger_{\mathrm{T,K'},\sigma}(\boldsymbol{k}_1) \hat{e}_{\mathrm{B,K},\sigma}(\boldsymbol{k}_2)], \tag{S39}$$

$$\hat{\mathcal{F}}^{(h)}_{\boldsymbol{k}_1,\boldsymbol{k}_2} \equiv \sum_\sigma [\hat{e}^\dagger_{\mathrm{T,K},\sigma}(\boldsymbol{k}_1) \hat{e}_{\mathrm{B,K'},\sigma}(\boldsymbol{k}_2) + \hat{e}^\dagger_{\mathrm{B,K},\sigma}(\boldsymbol{k}_1) \hat{e}_{\mathrm{T,K'},\sigma}(\boldsymbol{k}_2)], \tag{S40}$$

where the hole creation operator is understood as $\hat{h}^\dagger_{\mathrm{T/B},\sigma}(\boldsymbol{q}) \equiv \hat{e}_{\mathrm{T/B},v,\bar{\sigma}}(-\boldsymbol{q})$. The parameter V_a is determined by the Coulomb potential at $|\boldsymbol{K}-\boldsymbol{K}'|$ $(2\pi e^2/\varepsilon|\boldsymbol{K}-\boldsymbol{K}'|$, see also Refs. [24, 25]) and, since both scattered particles switch layers, by the corresponding wave function overlaps. Given the small interlayer separation $l \simeq 0.6\,\mathrm{nm}$ and strong hole tunneling (on the order of tens of meV), these overlaps can be non-negligible. In our approach, V_a is a phenomenological parameter that is further assumed to be momentum-independent.

In what follows, we demonstrate that Eq. (S38) leads to the intervalley interlayer exciton hybridization of the form:

$$\hat{H}_{\mathrm{intervalley}} = \hat{\mathcal{W}}_{\mathrm{inter}} \hat{X}^\dagger_{\mathrm{B,K}} \hat{X}_{\mathrm{T,K'}} + \mathrm{h.c.} \tag{S41}$$

We evaluate the strength $\hat{\mathcal{W}}_{\mathrm{inter}}$ within second-order perturbation theory, cf. Eq. (S49), and show that its mean expectation value can be nonzero $\langle \hat{\mathcal{W}}_{\mathrm{inter}} \rangle \neq 0$ even if the system does not spontaneously break any of the symmetries mentioned in Sec. X, thereby providing an interpretation of the weak intensity asymmetry in, for example, Fig. S10, as well as optical size effects discussed in Sec. V.

A. Anomalous terms and the U(1) layer symmetry

Before we proceed, let us mention that strictly speaking, the Hamiltonian in Eq. (S38) contains terms such as $\sim \hat{e}^\dagger_{\mathrm{T,K}} \hat{e}^\dagger_{\mathrm{T,K'}} \hat{e}_{\mathrm{B,K}} \hat{e}_{\mathrm{B,K'}}$, which do not conserve the total number of particles in the top or bottom layers, explicitly breaking the U(1) layer symmetry. To illustrate this, we write for these processes a similar reduced expression as in

Eq. (S20) (as in Sec. X, we disregard the electron spin):

$$\frac{V_a}{\mathcal{A}}\sum_{\boldsymbol{k},\boldsymbol{k}'}\Big\{\hat{\Delta}_x(\boldsymbol{k})\hat{\Delta}_x(\boldsymbol{k}')+\hat{\Delta}_y(\boldsymbol{k})\hat{\Delta}_y(\boldsymbol{k}')+\hat{\Delta}_z(\boldsymbol{k})\hat{\Delta}_z(\boldsymbol{k}')-\hat{\Delta}_0(\boldsymbol{k})\hat{\Delta}_0(\boldsymbol{k}')+\text{h.c.}\Big\}. \tag{S42}$$

Clearly, these anomalous terms in Eq. (S42) break the U(1) layer symmetry.

Apart from the processes in Fig. S16, anomalous terms generally appear in the Hamiltonian through the density-density Coulomb interactions and have the structure $\hat{e}^\dagger_{\mathrm{T},\alpha}\hat{e}^\dagger_{\mathrm{T},\beta}\hat{e}_{\mathrm{B},\gamma}\hat{e}_{\mathrm{B},\delta}$. These terms, involving electron layer switching (with electron density operators of the form $\hat{e}^\dagger_{\mathrm{T},\alpha}\hat{e}_{\mathrm{B},\beta}$), are expected to be suppressed due to wave function overlap considerations. Assuming the AQN is a good quantum number – supported by the small Fermi momentum relative to the lattice scale momentum, and experimentally by the absence of electron tunneling [16] – the $\mathcal{C}_3$-symmetry imposes that the anomalous terms take the form $\hat{e}^\dagger_{\mathrm{T,K}}\hat{e}^\dagger_{\mathrm{T,K}}\hat{e}_{\mathrm{B,K'}}\hat{e}_{\mathrm{B,K'}}$, $\hat{e}^\dagger_{\mathrm{T,K'}}\hat{e}^\dagger_{\mathrm{T,K'}}\hat{e}_{\mathrm{B,K}}\hat{e}_{\mathrm{B,K}}$, or $\hat{e}^\dagger_{\mathrm{T,K}}\hat{e}^\dagger_{\mathrm{T,K'}}\hat{e}_{\mathrm{B,K'}}\hat{e}_{\mathrm{B,K}}$. For the first two types of terms, the involved density operators have intervalley character such as $\hat{e}^\dagger_{\mathrm{T,K}}\hat{e}_{\mathrm{B,K'}}$, so that the involved Coulomb processes carry a large momentum of the order $\boldsymbol{K}-\boldsymbol{K}'$. Therefore, we expect that the corresponding anomalous terms are weak compared to the primary Coulomb interactions in Eq. (S14). Indeed, a similar estimate as in Sec. X for $n = 2\times10^{12}\,\mathrm{cm}^{-2}$ gives a value $\sim V_a n/4 \simeq 1\,\mathrm{meV}$, which is about two orders of magnitude weaker than in Sec. X A if we substitute $V_a = 2\pi e^2/\varepsilon|\boldsymbol{K}-\boldsymbol{K}'|$, i.e., if we neglect the wave-function overlaps that might further suppress this estimate. For the third direct processes of the form $\hat{e}^\dagger_{\mathrm{T,K}}\hat{e}^\dagger_{\mathrm{T,K'}}\hat{e}_{\mathrm{B,K'}}\hat{e}_{\mathrm{B,K}}$, these interactions do not necessarily occur at large momenta near $\boldsymbol{K}-\boldsymbol{K}'$. However, the corresponding density operators at a finite momentum $\boldsymbol{q}$ have contributions from electronic states with different AQNs, such as $\hat{e}^\dagger_{\mathrm{T,K}}(\boldsymbol{k}+\boldsymbol{q})\hat{e}_{\mathrm{B,K}}(\boldsymbol{k})$. Due to this AQN mismatch for electrons in the same valley but opposite layers, these contributions should vanish for $q\to0$ (otherwise, electron tunneling would be allowed, in disagreement with the experiment of Ref. [16]). Hence, the anomalous terms from direct interactions are also expected to be suppressed in the low-density regime.

We finally note that the anomalous terms break the U(1) symmetry down to $\mathbb{Z}_2$, i.e., the Hamiltonian remains symmetric under $\hat{\Delta}_a\to-\hat{\Delta}_a$. Therefore, a local expectation value $\langle\hat{\Delta}_a\rangle\neq0$ breaks this $\mathbb{Z}_2$ symmetry. This implies that even if there are anomalous terms, which are not suppressed, we get order parameter fluctuations that involve not only the magnitude of $\langle\hat{\Delta}_a\rangle\neq0$ but also its sign. As in Sec. X, we, thus, expect such fluctuations to manifest in the stochastic anti-crossing.

B. Second-order perturbation theory

We write the exciton wave function as:

$$\hat{X}^\dagger_{\mathrm{T,K'}}(\boldsymbol{k})=\frac{1}{\sqrt{\mathcal{A}}}\sum_{\boldsymbol{q}}\Psi_X(\boldsymbol{q})\hat{e}^\dagger_{\mathrm{T}}(\boldsymbol{K}'+\boldsymbol{q}+\boldsymbol{k}/2)\hat{h}^\dagger_{\mathrm{B}}(-\boldsymbol{K}'-\boldsymbol{q}+\boldsymbol{k}/2), \tag{S43}$$

where $\boldsymbol{k}$ ($\boldsymbol{q}$) is the $X_{\mathrm{T,K'}}$-exciton center-of-mass momentum (momentum of the relative motion). In TMDs, the binding energy of this state is in the hundreds of meV range, and the Bohr radius a_X is on the order of a few nanometers. Consequently, the momenta of both the electron and hole of this exciton are large, much larger than the Fermi momentum k_F. From the perspective of the $X_{\mathrm{T,K'}}$-state, the hole-flip process scatters the $X_{\mathrm{T,K'}}$-exciton into an electron-hole pair, with the hole now belonging to the top K-valley. Both particles are expected to have large momenta on the order of a_X^{-1}. We remark that the form in Eq. (S43) is only approximate, as i) the $X_{\mathrm{T,K'}}$-state is expected to have appreciable spectral weight also in the K-valley and ii) the exciton linewidth is rather large $\gamma\simeq10\,\mathrm{meV}$ in MoS_2-homobilayers [26].

From the energy perspective, it seems most intuitive to understand the hole-flip Hamiltonian as if it couples the $\hat{X}_{\mathrm{T,K'}}$-exciton to the lowest-energy momentum-indirect exciton $\hat{Y}_{\mathrm{T,K'}}$ at the cost of perturbing the two involved Fermi seas. This excitonic $\hat{Y}_{\mathrm{T,K'}}$-state should have properties similar to the A-excitons, except it is optically dark. More generally, we will consider not only the lowest-energy intralayer state but the entire Rydberg series, encompassing both bound and scattering states and fully covering the phase space of the involved electron-hole pair. The Rydberg states are expressed as:

$$\hat{Y}^\dagger_{\mathrm{T,K'},\nu}(\boldsymbol{k})=\frac{1}{\sqrt{\mathcal{A}}}\sum_{\boldsymbol{q}}\Psi_\nu(\boldsymbol{q})\hat{e}^\dagger_{\mathrm{T}}(\boldsymbol{K}'+\boldsymbol{q}+\boldsymbol{k}/2)\hat{h}^\dagger_{\mathrm{T}}(-\boldsymbol{K}-\boldsymbol{q}+\boldsymbol{k}/2). \tag{S44}$$

Here, the index ν runs over the entire Rydberg series, while $\boldsymbol{k}$ is the center-of-mass momentum – the Rydberg series of the two-dimensional hydrogen atom is known analytically [27–30].

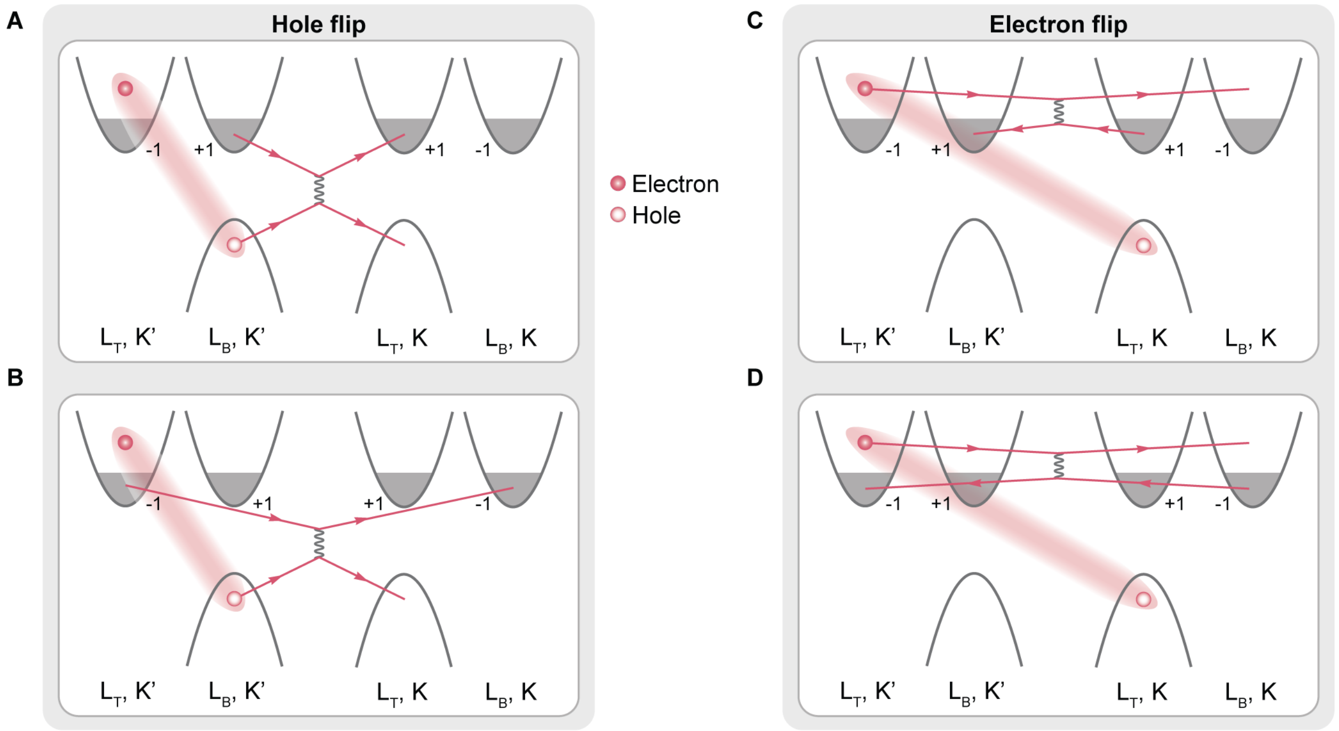

FIG. S16. The hole flip (left) occurs through a scattering process, where the hole of the $X_{\mathrm{T,K'}}$-state transitions from the bottom K'-valley to the top K-valley, accompanied by one of the two depicted Fermi sea electron scatterings. Note that when an electron scatters, its spin and AQN are both preserved. The electron flip (right) occurs similarly: the electron of the intermediate $Y_{\mathrm{T,K'}}$-state transitions from the top K'-valley to the bottom K-valley, accompanied by one of the two conduction-band electron scatterings.

The hole-flip matrix elements can then be written as:

$$\hat{\mathcal{U}}(\boldsymbol{p},\nu;\boldsymbol{p}') \equiv \langle Y_{\mathrm{T,K'},\nu}(\boldsymbol{p})|\,\hat{H}_{\mathrm{int}}\,|X_{\mathrm{T,K'}}(\boldsymbol{p}')\rangle = -\frac{V_a}{\mathcal{A}}\mathcal{J}_\nu(\boldsymbol{p},\boldsymbol{p}')\sum_{\boldsymbol{q}}\hat{\mathcal{F}}^{(h)}_{\boldsymbol{q}+\boldsymbol{p}',\boldsymbol{q}+\boldsymbol{p}}, \tag{S45}$$

where

$$\mathcal{J}_\nu(\boldsymbol{p},\boldsymbol{p}') = \frac{1}{\mathcal{A}}\sum_{\boldsymbol{k}}\Psi^*_\nu(\boldsymbol{p}'-\boldsymbol{k}-\boldsymbol{p}/2)\Psi_X(\boldsymbol{p}'/2-\boldsymbol{k}). \tag{S46}$$

Similarly, the electron-flip matrix elements are given by:

$$\hat{\mathcal{V}}(\boldsymbol{p}'';\boldsymbol{p}',\nu) \equiv \langle X_{\mathrm{B,K}}(\boldsymbol{p}'')|\,\hat{H}_{\mathrm{int}}\,|Y_{\mathrm{T,K'},\nu}(\boldsymbol{p}')\rangle = \frac{V_a}{\mathcal{A}}\tilde{\mathcal{J}}_\nu(\boldsymbol{p}'',\boldsymbol{p}')\sum_{\boldsymbol{k}}\hat{\mathcal{F}}^{(e)}_{\boldsymbol{k}-\boldsymbol{p}'',\boldsymbol{k}-\boldsymbol{p}'}, \tag{S47}$$

where

$$\tilde{\mathcal{J}}_\nu(\boldsymbol{p}'',\boldsymbol{p}') = \frac{1}{\mathcal{A}}\sum_{\boldsymbol{k}}\Psi^*_X(\boldsymbol{k}-\boldsymbol{p}'+\boldsymbol{p}''/2)\Psi_\nu(\boldsymbol{k}-\boldsymbol{p}'/2). \tag{S48}$$

Within second-order perturbation theory, Eqs. (S45) and (S47) result in the following effective interlayer exciton hybridization of the bright excitons with $\boldsymbol{p}'=\boldsymbol{p}''=\boldsymbol{0}$:

$$\hat{\mathcal{W}}_{\mathrm{inter}} \approx -\frac{V_a^2}{\mathcal{A}^2}\sum_{\boldsymbol{p},\nu}\sum_{\boldsymbol{k},\boldsymbol{q}}\frac{\tilde{\mathcal{J}}_\nu(\boldsymbol{0},\boldsymbol{p})\mathcal{J}_\nu(\boldsymbol{p},\boldsymbol{0})}{E_X(\boldsymbol{0})-E_\nu(\boldsymbol{p})+i\gamma_\nu}\hat{\mathcal{F}}^{(e)}_{\boldsymbol{q},\boldsymbol{q}-\boldsymbol{p}}\hat{\mathcal{F}}^{(h)}_{\boldsymbol{k},\boldsymbol{k}+\boldsymbol{p}}, \tag{S49}$$

where $E_\nu(\boldsymbol{p}) = E_\nu + p^2/(2M_Y)$, $E_X(\boldsymbol{p}') = E_X + p'^2/(2M_X)$, and $\gamma_\nu(n,T)$ represents the decay rate of the intermediate state. We neglected the energy correction to the denominator coming from perturbing the involved Fermi seas – such

correction is expected to be small as it is set by the Fermi energy, which is typically much smaller than the energy detuning $|\delta_\nu|$, $\delta_\nu = E_\nu - E_X$. For a similar reason, the entire momentum dependence of the denominator can be disregarded. We note that the electron flip can occur first, followed by the hole flip, and the corresponding expression can be computed using the same analysis as outlined above. It is instructive to rewrite Eq. (S49) in the reduced form as in Eq. (S21) using the order parameter operators (S19) (again, we disregard the electron spin):

$$\begin{aligned}\hat{\mathcal{W}}_{\text{inter}} \to &- V_a^2 \int \frac{d^2\boldsymbol{k}}{(2\pi)^2} \int \frac{d^2\boldsymbol{k}'}{(2\pi)^2} \sum_\nu \frac{\tilde{\mathcal{J}}_\nu(\boldsymbol{0},\boldsymbol{0})\mathcal{J}_\nu(\boldsymbol{0},\boldsymbol{0})}{E_X - E_\nu + i\gamma_\nu} \Big\{ 2[\hat{\Delta}_x^\dagger(\boldsymbol{k})\hat{\Delta}_x(\boldsymbol{k}') + \hat{\Delta}_y^\dagger(\boldsymbol{k})\hat{\Delta}_y(\boldsymbol{k}')] \\ &+ [\hat{\Delta}_x^\dagger(\boldsymbol{k})\hat{\Delta}_x^\dagger(\boldsymbol{k}') + \hat{\Delta}_y^\dagger(\boldsymbol{k})\hat{\Delta}_y^\dagger(\boldsymbol{k}')] + [\hat{\Delta}_x(\boldsymbol{k})\hat{\Delta}_x(\boldsymbol{k}') + \hat{\Delta}_y(\boldsymbol{k})\hat{\Delta}_y(\boldsymbol{k}')] \Big\} \\ &- V_a^2 \int \frac{d^2\boldsymbol{k}}{(2\pi)^2} \int \frac{d^2\boldsymbol{k}'}{(2\pi)^2} \sum_\nu \frac{\tilde{\mathcal{J}}_\nu(\boldsymbol{0},\boldsymbol{k}-\boldsymbol{k}')\mathcal{J}_\nu(\boldsymbol{k}-\boldsymbol{k}',\boldsymbol{0})}{E_X - E_\nu + i\gamma_\nu} \Big\{ - [\hat{\Delta}_0(\boldsymbol{k}) - \hat{\Delta}_z(\boldsymbol{k})][\hat{\Delta}_0(\boldsymbol{k}') + \hat{\Delta}_z(\boldsymbol{k}')] \\ &- [\hat{\Delta}_0^\dagger(\boldsymbol{k}) - \hat{\Delta}_z^\dagger(\boldsymbol{k})][\hat{\Delta}_0^\dagger(\boldsymbol{k}') + \hat{\Delta}_z^\dagger(\boldsymbol{k}')] + \hat{n}_{\text{B,K}'}(\boldsymbol{k})(1 - \hat{n}_{\text{T,K}}(\boldsymbol{k}')) + \hat{n}_{\text{T,K}'}(\boldsymbol{k})(1 - \hat{n}_{\text{B,K}}(\boldsymbol{k}')) \Big\},\end{aligned} \tag{S50}$$

where $\hat{n}_{\text{T,K}}(\boldsymbol{k}) \equiv \hat{e}_{\text{T,K}}^\dagger(\boldsymbol{k})\hat{e}_{\text{T,K}}(\boldsymbol{k})$, etc.

The expression in Eq. (S50) suggests that the expectation value $\langle\hat{\mathcal{W}}_{\text{inter}}\rangle$ can be nonzero in the regime when the magnitude of the order parameter has developed but the phase is fluctuating. This arises because the coupling in Eq. (S50) contains terms determined solely by the magnitude of the order parameter, in contrast to the intravalley interlayer exciton hybridization in Eq. (S21), which depends on the relative phase between the hole tunneling and the spatially fluctuating interlayer coherence order parameter. Additionally, the last two terms in Eq. (S50) indicate that finite hybridization can occur even in a Fermi liquid state. We note, however, that the coupling in Eq. (S50) is expected to be small due to the factor V_a^2, consistent with the experimental observations in Fig. 2E,F of the main text.

[1] A. Singh and A. K. Singh, Origin of n-type conductivity of monolayer MoS_2, Phys. Rev. B **99**, 121201 (2019).
[2] Y. Park, N. Li, D. Jung, L. T. Singh, J. Baik, E. Lee, D. Oh, Y. D. Kim, J. Y. Lee, J. Woo, *et al.*, Unveiling the origin of n-type doping of natural MoS_2: carbon, npj 2D Mater. Appl. **7**, 60 (2023).
[3] C. Gong, L. Colombo, R. M. Wallace, and K. Cho, The unusual mechanism of partial Fermi level pinning at metal–MoS_2 interfaces, Nano Lett. **14**, 1714 (2014).
[4] J. Kang, W. Liu, D. Sarkar, D. Jena, and K. Banerjee, Computational study of metal contacts to monolayer transition-metal dichalcogenide semiconductors, Phys. Rev. X **4**, 031005 (2014).
[5] D. Tebbe, M. Schütte, K. Watanabe, T. Taniguchi, C. Stampfer, B. Beschoten, and L. Waldecker, Tailoring the dielectric screening in WS_2–graphene heterostructures, npj 2D Mater. Appl. **7**, 29 (2023).
[6] P. E. Dolgirev, J. Marino, D. Sels, and E. Demler, Non-Gaussian correlations imprinted by local dephasing in fermionic wires, Phys. Rev. B **102**, 100301 (2020).
[7] L. Sponfeldner, N. Leisgang, S. Shree, I. Paradisanos, K. Watanabe, T. Taniguchi, C. Robert, D. Lagarde, A. Balocchi, X. Marie, *et al.*, Capacitively and inductively coupled excitons in bilayer MoS_2, Phys. Rev. Lett. **129**, 107401 (2022).
[8] T. Smoleński, P. E. Dolgirev, C. Kuhlenkamp, A. Popert, Y. Shimazaki, P. Back, X. Lu, M. Kroner, K. Watanabe, T. Taniguchi, *et al.*, Signatures of Wigner crystal of electrons in a monolayer semiconductor, Nature **595**, 53 (2021).
[9] G. Scuri, Y. Zhou, A. A. High, D. S. Wild, C. Shu, K. De Greve, L. A. Jauregui, T. Taniguchi, K. Watanabe, P. Kim, *et al.*, Large excitonic reflectivity of monolayer $MoSe_2$ encapsulated in hexagonal boron nitride, Phys. Rev. Lett. **120**, 037402 (2018).
[10] Y. Zhou, G. Scuri, D. S. Wild, A. A. High, A. Dibos, L. A. Jauregui, C. Shu, K. De Greve, K. Pistunova, A. Y. Joe, *et al.*, Probing dark excitons in atomically thin semiconductors via near-field coupling to surface plasmon polaritons, Nature Nanotechnol. **12**, 856 (2017).
[11] T. Hastie, R. Tibshirani, and J. Friedman, *The elements of statistical learning: data mining, inference, and prediction*, Vol. 2 (Springer, 2009).
[12] G. James, D. Witten, T. Hastie, R. Tibshirani, *et al.*, *An introduction to statistical learning*, Vol. 112 (Springer, 2013).
[13] L. Zheng, M. Ortalano, and S. D. Sarma, Exchange instabilities in semiconductor double-quantum-well systems, Phys. Rev. B **55**, 4506 (1997).
[14] J. Zhu and S. Das Sarma, Interaction and coherence in two-dimensional bilayers, Phys. Rev. B **109**, 085129 (2024).
[15] A. Stern, S. D. Sarma, M. P. Fisher, and S. Girvin, Dissipationless transport in low-density bilayer systems, Phys. Rev. Lett. **84**, 139 (2000).
[16] R. Pisoni, T. Davatz, K. Watanabe, T. Taniguchi, T. Ihn, and K. Ensslin, Absence of interlayer tunnel coupling of K-valley electrons in bilayer MoS_2, Phys. Rev. Lett. **123**, 117702 (2019).
[17] I. Spielman, J. Eisenstein, L. Pfeiffer, and K. West, Resonantly enhanced tunneling in a double layer quantum Hall ferromagnet, Phys. Rev. Lett. **84**, 5808 (2000).

[18] K. A. Lin, N. Prasad, G. W. Burg, B. Zou, K. Ueno, K. Watanabe, T. Taniguchi, A. H. MacDonald, and E. Tutuc, Emergence of interlayer coherence in twist-controlled graphene double layers, Phys. Rev. Lett. **129**, 187701 (2022).
[19] X.-G. Wen and A. Zee, Tunneling in double-layered quantum Hall systems, Phys. Rev. B **47**, 2265 (1993).
[20] A. Stern, S. M. Girvin, A. H. MacDonald, and N. Ma, Theory of interlayer tunneling in bilayer quantum Hall ferromagnets, Phys. Rev. Lett. **86**, 1829 (2001).
[21] M. M. Fogler and F. Wilczek, Josephson effect without superconductivity: realization in quantum Hall bilayers, Phys. Rev. Lett. **86**, 1833 (2001).
[22] S. M. Girvin and K. Yang, *Modern condensed matter physics* (Cambridge University Press, 2019).
[23] C. B. Hanna, D. Haas, and J. C. Díaz-Vélez, Double-layer systems at zero magnetic field, Phys. Rev. B **61**, 13882 (2000).
[24] R. A. Żak, D. L. Maslov, and D. Loss, Ferromagnetic order of nuclear spins coupled to conduction electrons: A combined effect of electron-electron and spin-orbit interactions, Phys. Rev. B **85**, 115424 (2012).
[25] D. Miserev, J. Klinovaja, and D. Loss, Exchange intervalley scattering and magnetic phase diagram of transition metal dichalcogenide monolayers, Phys. Rev. B **100**, 014428 (2019).
[26] S. Helmrich, K. Sampson, D. Huang, M. Selig, K. Hao, K. Tran, A. Achstein, C. Young, A. Knorr, E. Malic, *et al.*, Phonon-assisted intervalley scattering determines ultrafast exciton dynamics in $MoSe_2$ bilayers, Phys. Rev. Lett. **127**, 157403 (2021).
[27] C. Y.-P. Chao and S. L. Chuang, Analytical and numerical solutions for a two-dimensional exciton in momentum space, Phys. Rev. B **43**, 6530 (1991).
[28] X. Yang, S. Guo, F. Chan, K. Wong, and W. Ching, Analytic solution of a two-dimensional hydrogen atom. I. Nonrelativistic theory, Phys. Rev. A **43**, 1186 (1991).
[29] D. Parfitt and M. Portnoi, The two-dimensional hydrogen atom revisited, J. Math. Phys. **43**, 4681 (2002).
[30] D. K. Efimkin, E. K. Laird, J. Levinsen, M. M. Parish, and A. H. MacDonald, Electron-exciton interactions in the exciton-polaron problem, Phys. Rev. B **103**, 075417 (2021).
